# Metal-free sulfur-doped reduced graphene oxide electrocatalysts for promising production of hydrogen peroxide: construction and identification of active sites†

**DOI:** 10.1039/d5sc03069b

**Published:** 2025-06-02

**Authors:** Sifan Li, Shiwen Du, Jiansheng Li, Wenjun Fan, Yang Yang, Peng Zhao, Haotian Zhu, Wansheng You, Xiaojing Sang, Fuxiang Zhang

**Affiliations:** a School of Chemistry and Chemical Engineering, Liaoning Normal University Dalian 116029 Liaoning China lijiansheng@lnnu.edu.cn sangxj923@nenu.edu.cn; b State Key Laboratory of Catalysis, Dalian Institute of Chemical Physics, Chinese Academy of Sciences Dalian 116023 Liaoning China fxzhang@dicp.ac.cn; c School of Physics and Materials Engineering, Dalian Minzu University Dalian 116023 Liaoning China; d Department of Biochemical Engineering, Chaoyang Normal University Chaoyang 122000 Liaoning China

## Abstract

Identifying and tailoring active sulfur configurations in heteroatom-doped carbon electrocatalysts for the selective 2e^−^ oxygen reduction reaction (ORR) pathway remains a significant challenge. Here we designed and synthesized sulfur-doped reduced graphene oxide electrocatalysts containing C–S and C–SO_*x*_ moieties (denoted as S_*x*_RGO, *x* = 1, 10, 20) for promising ORR into hydrogen peroxide (H_2_O_2_). The optimized S_10_RGO catalyst exhibits unexpected H_2_O_2_ selectivity of *ca.* 90% across a wide voltage range of 0.10–0.65 V, accompanied with excellent long-term stability (40 h) in an alkaline flow cell with 90.5% H_2_O_2_ faradaic efficiency at an industrial current density of 300 mA cm^−2^. Theoretical and experimental analyses integrally reveal and identify the C–S and C–SO_*x*_ groups as the main active sites in the carbon-based catalyst. Specifically, the C–S group is found to favor the formation of OOH*, while the C–SO_*x*_ group not only facilitates the desorption of OOH* but also modulates interfacial mass transport kinetics, thereby creating a favorable microenvironment for H_2_O_2_ generation.

## Abstract 2

C–S/C–SO_*x*_ groups regulate *OOH intermediates *via* adsorption–desorption, enabling non-metallic carbon catalysts for efficient 2e^−^ ORR at high current densities in alkaline media.

## Graphical abstract

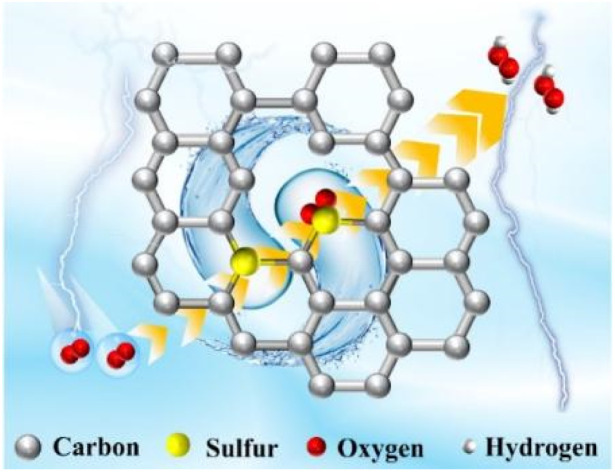

## 1 Introduction

Hydrogen peroxide (H_2_O_2_), as a green oxidizer and potential liquid fuel, has been extensively used in the fields of environmental remediation, chemical synthesis and energy conversion.^1–4^ Currently, the large-scale production of H_2_O_2_ relies on the energy and waste-intensive anthraquinone process, suffering from high cost.^5,6^ In contrast, the electrocatalytic two-electron oxygen reduction reaction (2e^−^ ORR) offers a sustainable alternative for onsite H_2_O_2_ production.^7^ To date, precious metals such as Pt, Au, Pd and their alloys, along with non-precious metal catalysts such as Fe, Co, and Ni have been reported to exhibit remarkable selectivity towards the 2e^−^ ORR.^8–11^ However, considering the scarcity of precious metals and the potential solubility of non-precious metals, significant efforts have been dedicated to the development of metal-free heteroatom doped carbon-based materials due to their adjustable surface and electronic structure properties.^12–14^

Sulfur-doping is particularly intriguing among various metal-free dopants (B, N, O, *etc.*) owing to its unique advantages including the larger atomic radius and electron-rich characteristics.^15–18^ Its incorporation not only induces spin density redistribution in the carbon skeleton but also enhances O_2_ adsorption by elevating electron density, thereby reducing the overpotential for the ORR. Basically, most studies on sulfur-doped carbon materials have focused on 4e^−^ ORR activity instead of 2e^−^ ORR.^19–21^ While some recent works reveal that sulfur co-doping with other heteroatoms (*e.g.*, N, F) or defects can lead to a 2e^−^ ORR process, these findings often highlight the role of sulfur atoms in modulating the electronic structure of other atoms, and the intrinsic role of sulfur moieties in the 2e^−^ ORR remains under-investigated.^22,23^ The design and fabrication of efficient and stable sulfur-doped carbon materials, specifically tailored for selective 2e^−^ ORR and capable of withstanding high current densities, continues to pose a significant challenge. Moreover, in the limited reports on the 2e^−^ ORR behavior of sulfur-doped carbon materials, the construction and identification of distinct sulfur configurations, such as C–S, C–SO_*x*_, S–H, and S–S, as well as the elucidation of the structure–activity relationship, remain underexplored. This is a demanding yet critical endeavor for the advancement of catalyst performance and selectivity.^24–30^ Additionally, conventional studies have primarily focused on electronic structure engineering through heteroatom doping, often overlooking the potential impact on surface chemical microenvironments. Notably, recent studies have shown that adding dimethyl sulfoxide (DMSO) to electrolytes can enhance H_2_O_2_ selectivity by forming H_2_O–DMSO hydrogen-bonding networks, which alters proton transfer kinetics and facilitates H_2_O_2_ production.^31^ This insight provides a new perspective for understanding the electrocatalytic 2e^−^ ORR process of S-doped carbon materials.

In this work, a series of precisely engineered C–S and C–SO_*x*_ co-modified carbon-based catalysts (S_*x*_RGO) were synthesized using graphene oxide (GO) and sulfur as precursors. The optimized S_10_RGO catalyst shows superior electrocatalytic H_2_O_2_ performance to most previously reported sulfur-doped carbon materials. Combined with density functional theory (DFT) calculations, a significant correlation between sulfur configurations (C–S and C–SO_*x*_) and the 2e^−^ ORR catalytic performance was built, in which the C–S group was disclosed to favor the formation of the key oxygenated intermediate OOH*, while the C–SO_*x*_ group was shown to facilitate the desorption of OOH*. In addition, the kinetic effects of surface SO_*x*_ groups on H_2_O_2_ generation were studied. This work delivers the first report on the remarkable influence of distinct sulfur configurations (*e.g.*, S–C, SO_*x*_) on intermediate adsorption and H_2_O_2_ generation kinetics, which provides a clearer view and a new perspective for understanding the selectivity and activity of sulfur-doped carbon materials in the ORR, and lays a foundation for designing high-performance electrocatalysts and establishing precise control over multi-electron reaction selectivity.

## 2 Experimental

### Synthesis of S_*x*_RGO

Before use, GO was subjected to a series of purification steps, including washing with a 10 wt% hydrochloric acid solution, followed by rinsing with deionized water and acetone, in order to remove metal impurities. Subsequently, it was dried overnight at 40 °C in an oven. GO and sulfur sublimated powder with various mass ratios (*e.g.*, 1 : 10 for S_10_RGO) was initially ground thoroughly in an agate mortar. The prepared mixed powders were then collected into a porcelain boat and placed at the center of a tube furnace. By continuously feeding Ar gas (50 sccm), the furnace was heated up to 160 °C at a ramp rate of 2 °C min^−1^. After keeping this temperature for 6 h, the annealing temperature was increased to 500 °C with a ramp rate of 5 °C min^−1^ and held there for 1 h, followed by natural cooling under a continuous flow of Ar gas. The collected bright black product was denoted as S_*x*_RGO (*x* = 0, 1, 10, 20).

### Synthesis of S_10_RGO-500

S_10_RGO-500 was prepared *via* the same process as S_10_RGO except that the annealing temperature was directly raised to 500 °C with a ramp rate of 5 °C min^−1^ and held there for 1 h.

### Synthesis of S_0_RGO-160 and S_10_RGO-160

S_0_RGO-160 and S_10_RGO-160 were prepared *via* the same process as S_0_RGO and S_10_RGO except that the annealing temperature was raised up to 160 °C with a ramp rate of 2 °C min^−1^ and held there for 6 h.

### Rotation ring disk electrode (RRDE) test

The electrochemical experiments were performed on a CHI 760E electrochemical workstation utilizing a three-electrode system. An RRDE (Taizhou Keruite Analytical Instrument, Co., Ltd, disk area: 0.1256 cm^2^) with a Pt ring (ring area: 0.1664 cm^2^) was used as the working electrode, a graphite rod as the counter electrode and an Ag/AgCl electrode as the reference electrode, respectively. To prepare the catalyst ink, 3.0 mg of the obtained catalysts were mixed in 1 mL of a solution containing 980 μL of deionized water/ethanol mixed solution (*V*_water_/*V*_ethanol_ = 5 : 1) and 20 μL of 5 wt% Nafion solution. The mixture was then subjected to ultrasonic treatment for 60 min to form homogeneous inks. Before measurement, the RRDE was polished with 0.30, 0.10 and 0.05 μm alumina powders (Chenhua) and then cleaned with deionized water. Subsequently, 6 μL (a loading of roughly 0.143 mg cm^−2^) of the catalyst ink was drop-cast onto a disk electrode of the RRDE tip spinning at an initial rate of 150 rpm and advanced to 300 rpm to achieve uniform electrode coverage. Two electrolytes with pH ∼13 (0.1 M KOH) and ∼7 (0.1 M Na_2_SO_4_) were used at room temperature. All potentials measured against an Ag/AgCl electrode were converted to the reversible hydrogen electrode (RHE). The H_2_O_2_ productivity and selectivity were determined through linear sweep voltammetry (LSV) under O_2_-saturated conditions, with a scan rate of 10 mV s^−1^ at 1600 rpm, within the potential range of 0 V to 0.90 V, while maintaining the platinum ring electrode potential at 1.30 V. The collection efficiency (*N*) was determined to be 0.43 by the redox reaction of [Fe(CN)_6_]^4−^/[Fe(CN)_6_]^3−^ according to the reported procedures.^32^ The double-layer capacitance was determined from cyclic voltammograms in the non-faradaic region at different scan rates (20, 40, 60, 80 and 100 mV s^−1^). A chronoamperometry test was performed at a disk electrode potential of 0.52 V in O_2_-saturated 0.1 M KOH electrolyte with an RRDE rotating speed of 1600 rpm. The Pt ring electrode was cleaned by rapid CV scanning from 0 V to −0.3 V and the electrolyte was refreshed every 2 h during the continuous operation. The measured potentials using a three-electrode setup of RRDE have no *iR* compensation.

Experimental details are described in the ESI.†

### H_2_O_2_ concentration measurement

The H_2_O_2_ concentrations in the electrolytes were measured by a Ce^4+^ titration method based on the following equation:

1 H_2_O_2_ + 2Ce(SO_4_)_2_ → Ce_2_(SO_4_)_3_ + H_2_SO_4_ + O_2_

The reaction of the yellow-colored Ce^4+^ to the colorless Ce^3+^ proceeds in the presence of H_2_O_2_. Thus, the concentration of Ce^4+^ can be measured by UV-vis absorption spectroscopy. A typical calibration curve was plotted by linear fitting of the absorbance values at 319 nm for Ce^4+^.

The faradaic efficiency (FE) for H_2_O_2_ generation was calculated as follows:

2 FE% = (*C* × *V* × *F* × 2)/(*i* × *t*) × 100%

where *C*, *V*, *F*, *i*, and *t* are the produced H_2_O_2_ concentration (mol L^−1^) in the electrolyte, the volume of electrolyte (L), Faraday constant (96 485 C mol^−1^), the operating current (A) and the test time (s), respectively.

### Electrocatalytic performance in H-type cells

The bulk electrolysis measurements were conducted with a typical H-type cell, which contains 30 mL electrolyte (0.1 M KOH) separated by a Nafion 117 membrane (Dupont), in which 10 mM EDTA was added to stabilize the produced H_2_O_2_. 200 μL catalyst ink was firstly coated on carbon paper (1 cm × 1 cm, Toray, TGPH090) with the loading of 600 μg cm^−2^. The graphite rod counter electrode was separated from the working electrode and Ag/AgCl reference electrode by a Nafion 117 membrane. The electrolyte in the cathode compartment was stirred with a stirring rate of 400 rpm to guarantee that the reactant can reach the electrode surface. Chronoamperometric processes were conducted to generate H_2_O_2_ in the O_2_-saturated electrolyte at 0.52 V. Bulk electrolysis for the H-type cell was carried out without *iR* compensation.

### Electrocatalytic performance in a flow cell

A flow cell was constructed to replicate the actual device, and the catalyst ink was coated onto the gas diffusion electrode (GDE, 1 × 3 cm^2^) by a spray gun as the cathode electrode, resulting in a loading mass of roughly 600 μg cm^−2^. The anode electrode was constructed using titanium felt, while the anode chamber and cathode chamber were separated by a Nafion 117 membrane. The electrolyte used was 1 M KOH, to which 10 mM EDTA was added for stabilizing the produced H_2_O_2_.^8^ The flow rate of the aqueous electrolyte was set at 8 mL min^−1^ by a peristaltic pump. High-purity O_2_ was continuously supplied through the opposite side of the catalyst with a flow rate of 30 mL min^−1^. LSV curves were performed at a scan rate of 10 mV s^−1^ and manually compensated by 100% *iR* effects. Electrochemical impedance spectroscopy (EIS) measurements under different biases were conducted in the flow cell in a frequency range from 0.01 Hz to 100 000 Hz with an AC amplitude of 5 mV. The electrolyte was updated before the EIS test for each system to avoid the impact of H_2_O_2_ accumulated in the solution during the EIS test.

### Computational details

All the density functional theory (DFT) calculations were performed with the aid of the Vienna *ab initio* simulation package (VASP) based on projector augmented wave (PAW) pseudopotentials with a cut-off energy of 500 eV.^33,34^ The generalized gradient approximation (GGA) with a Perdew–Burke–Ernzerhof (PBE) functional was employed to approximate the exchange and correlation effects during the relaxations.^35,36^ During structure optimizations, the convergence criterion of energy on each atom was set to 1.0 × 10^−5^ eV, while the force convergence threshold of each atom was set to 0.015 eV·Å^−1^. The models of RGO, SO_*x*_-RGO, S-RGO, and S/SO_*x*_-RGO were built with a vacuum spacing of 15 Å to simulate the ORR pathway using Monkhorst–Pack grids of 3 × 3 × 1.^37^ The Gibbs free energies of the ORR process were evaluated using the following equation:

3 *G* = *E*_DFT_ + *E*_ZPE_ − *TS*

where *E*_DFT_, *E*_ZPE_, and *TS* represent the DFT-calculated electronic energy, zero-point energy, and entropy of the system, respectively.

The details of materials and characterization are provided in the ESI.†

## 3 Results and discussion

### Synthesis and characterization

**Fig. 1:**
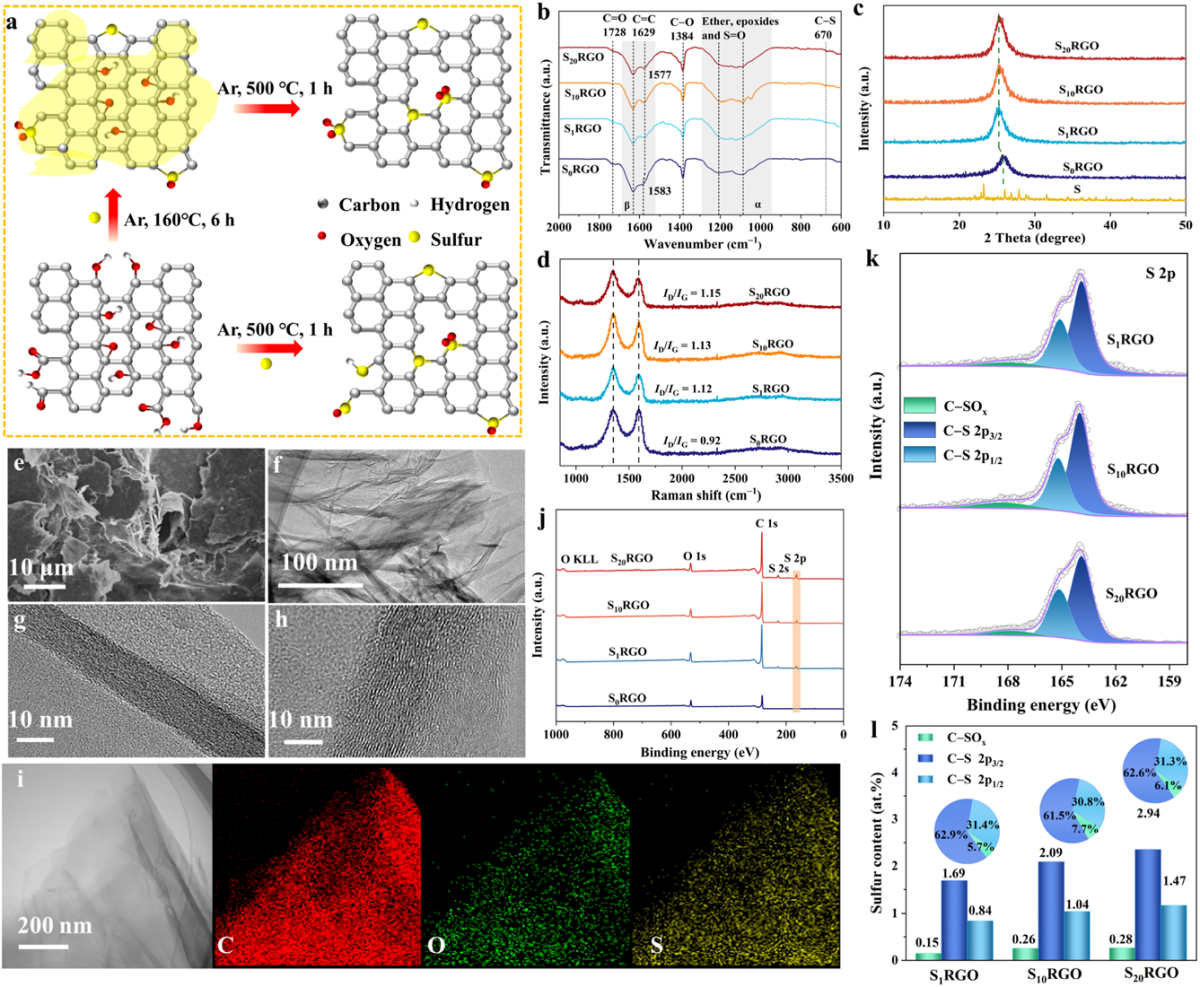
(a) Schematic illustration of the preparation of catalysts. (b) FTIR spectra, (c) XRD patterns, and (d) Raman spectra of S_*x*_RGO. (e) SEM and (f) TEM of S_10_RGO. HR-TEM images of (g) S_0_RGO and (h) S_10_RGO, and (i) element mapping of S_10_RGO. (j) Survey XPS spectra of S_*x*_RGO. (k) S 2p XPS spectra. (l) Proportional content of each sulfur configuration (inset is the sector diagram).

Two-step thermal annealing processes were conducted under an Ar atmosphere to obtain the target materials (S_*x*_RGO, “*x*” stands for the mass ratio of sublimed sulfur *versus* GO) using GO and sublimed sulfur as precursors. As shown in Fig. 1a, during the initial ramping step at 160 °C, molten sulfur infiltrates into the GO matrix and primarily reacts with the remaining oxygen-containing functional groups in GO, which may predominantly generate sulfur oxide species. Subsequently, when the annealing temperature is increased to 500 °C, the oxygen-containing functional groups were decomposed to induce disorders and vacancies in the GO lattice, which could serve as anchoring sites for sulfur atoms forming mainly C–S groups at this step (Fig. S1 and S2†).^38^ However, during the one-step thermal annealing process up to 500 °C, the final formed sulfur configurations include the S–H bond in addition to C–S and C–SO_*x*_ groups (Fig. S3†), which may result from the reaction of certain decomposed –COOH groups with sulfur under rapid ramping conditions. Here the doping state of S_*x*_RGO can be easily adjusted by regulating the programmed heating process.

Fourier transform infrared spectroscopy (FTIR), X-ray diffraction (XRD) and Raman spectroscopy were carried out to characterize the composition and structure of the as-obtained samples. As depicted in FTIR (Fig. 1b), the peak at 1728 cm^−1^ corresponds to the C=O stretching vibration for S_*x*_RGO (*x* = 0, 1, 10, 20). The peak shift of C=C from 1583 cm^−1^ for S_0_RGO gradually to 1577 cm^−1^ for S_*x*_RGO (*x* = 1, 10, 20, β-region) may come from the change in the surface chemistry of RGO due to sulfur doping.^39,40^ The peaks assigned to C–O–C (α-region) for S_0_RGO are shifted for S_*x*_RGO (*x* = 1, 10, 20), which should be derived from the introduction of sulfur oxide species that show overlapped peaks with ether and epoxides. Besides, it is worth noting that the peak corresponding to the C–S/C=S stretching vibration appears at 670 cm^−1^ for S_*x*_RGO (*x* = 1, 10, 20).^41^ XRD patterns (Fig. 1c) show a broad diffraction peak located at 2*θ* = 20–30° for all the S_*x*_RGO samples, which corresponds to the (002) facets of RGO.^16^ Compared to S_0_RGO, the (002) diffraction peak in S_*x*_RGO (*x* = 1, 10, 20) is prominently shifted to lower angles, suggesting a lattice expansion along the (002) direction. This result further confirms the successful doping of sulfur. Additionally, the absence of any crystalline sulfur peaks in the XRD patterns of S_*x*_RGO (*x* = 1, 10, 20) suggests that the doped sulfur is not present as crystal particles. This is consistent with the thermogravimetric results (Fig. S4†). Raman spectra (Fig. 1d and Table S1†) show that S_*x*_RGO (*x* = 1, 10, 20) give a higher intensity ratio of D and G bands to that of S_0_RGO (*I*_D_/*I*_G_ = 0.92), indicating increased number of defects in the carbon materials.^42^ Moreover, S_*x*_RGO (*x* = 1, 10, 20) show a much higher electrochemically active surface area (ECSA) with respect to the S_0_RGO, exposing many more active sites. Meanwhile, the S_*x*_RGO (*x* = 1, 10, 20) samples exhibit similar ECSAs, indicating their analogous surface roughness (Fig. S5 and Table S2†). The morphology structures of S_*x*_RGO (*x* = 0, 1, 10, 20) are similarly observed to exhibit wrinkled flakes and stacked layers according to their scanning electron microscope (SEM) and transmission electron microscope (TEM) images (Fig. 1e–i and S6–S9†). The high-resolution TEM (HR-TEM) images show that S_*x*_RGO (*x* = 1, 10, 20) display minimal distortion in both the basal and edge regions, while the apparent layer stripes indicate that the crystalline structure of RGO is maintained after the sulfur doping process, coinciding with the XRD and Raman results. The elemental mapping of S_*x*_RGO (*x* = 1, 10, 20) reveals a uniform distribution of sulfur within the RGO sheets, encompassing both the basal and edge regions.

The surface elements and the chemical states of S_*x*_RGO were detected by X-ray photoelectron spectroscopy (XPS) analysis (Table S3†). Apparently, sulfur signals were observed in the XPS survey spectra of S_*x*_RGO (*x* = 1, 10, 20) (Fig. 1j), indicating the successful incorporation of S species into RGO. No other elements than C, O, and S were detected. The atomic sulfur content in S_*x*_RGO (*x* = 1, 10, 20) samples increases proportionally with the increase of the S/GO mass ratio, which agrees with the results obtained from elemental analysis (Table S4†). The specific sulfur configuration in S_*x*_RGO was further analyzed using S 2p XPS spectra (Fig. 1k), in which three deconvoluted peaks were obtained with the binding energies located at 165.2 ± 0.1, 164 ± 0.1 and 167.5–170 eV, respectively. The first two peaks are assigned to the S 2p_1/2_ and S 2p_3/2_ of the C–S group, while the latter peak corresponds to the C–SO_*x*_ group.^43^ This result further proves the simultaneous construction of C–S and C–SO_*x*_ groups in the RGO, consistent with the FTIR results. According to their peak areas (Fig. 1l), the content of the C–S moiety is found to be gradually increased for S_1_RGO (2.53 at%), S_10_RGO (3.13 at%) and S_20_RGO (4.41 at%), while the content of the C–SO_*x*_ group is sharply improved from 0.15 at% for S_1_RGO to 0.26 at% for S_10_RGO, and then slightly rises to 0.28 at% for S_20_RGO. This interesting phenomenon was deduced to result from the fact that the C–SO_*x*_ group is primarily formed through the reaction of molten sulfur with the oxygen-containing functional groups at the sheet edges of GO during the initial annealing step at 160 °C, while the C–S moiety is generated from the anchoring of sulfur atoms by the vacancies in the bulk matrix of GO during the second annealing step. Besides, the contents of C–S and C–SO_*x*_ configurations in the S_*x*_RGO (*x* = 1, 10, 20) could also be revealed by analyzing the C 1s and O 1s XPS spectra (Fig. S10, S11 and Table S5†). It is worth noting that the configuration and content of sulfur-containing groups in the carbon material could be effectively adjusted through the programmed annealing process.

### Electrocatalytic ORR performances

**Fig. 2:**
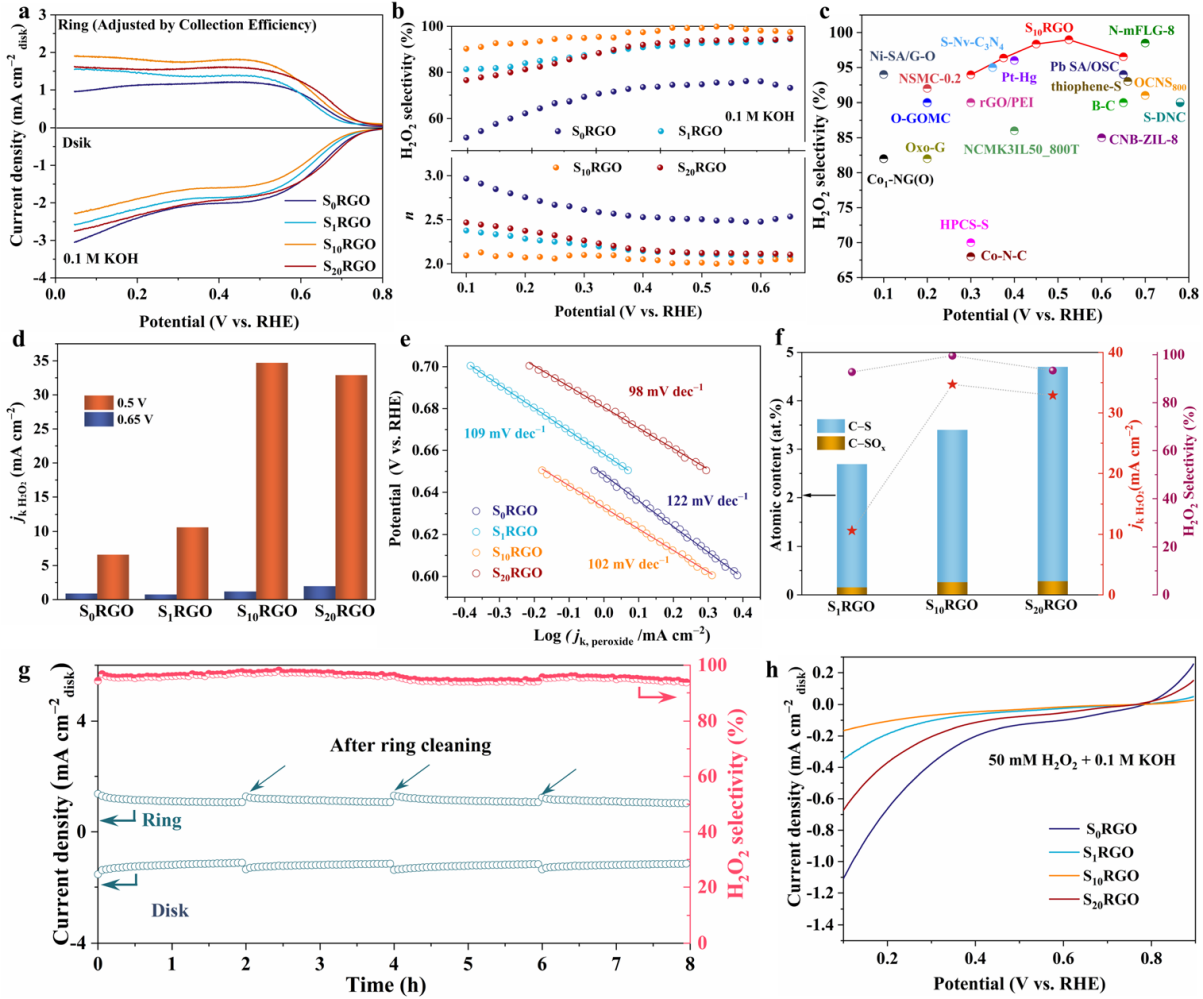
(a) LSV curves, (b) H_2_O_2_ selectivity and electron transfer number of S_*x*_RGO. (c) H_2_O_2_ selectivity on S_10_RGO and previously reported catalysts (as summarized in Table S6†) in alkaline solution. (d) Comparison of *j*_k_ at 0.5 V and 0.6 V. (e) Tafel plots. (f) Dependence of H_2_O_2_ selectivity and *j*_k_ on S moiety content. (g) Stability of S_10_RGO in 0.1 M KOH. (h) LSV curves of S_*x*_RGO in 0.1 M KOH containing 50 mM H_2_O_2_. Catalyst loading: 143 μg cm^−2^.

The electrocatalytic ORR performances of S_*x*_RGO were assessed *via* measuring the LSV curves with an RRDE as a working electrode at 1600 rpm in an O_2_-saturated 0.1 M KOH (Fig. S12–S14†). As shown in Fig. 2a, the larger ring current of S_*x*_RGO (*x* = 1, 10, 20) compared to that of S_0_RGO indicates the critical role of sulfur moieties in enhancing 2e^−^ ORR activity. Fig. 2b presents the H_2_O_2_ selectivity and electron transfer number (*n*) of S_*x*_RGO. Notably, the S_10_RGO exhibits an H_2_O_2_ selectivity of over 90% and an electron transfer number below 2.2 in a wide potential range from 0.10 to 0.65 V. It delivers a maximum H_2_O_2_ selectivity of 98.9% and a minimum *n* value of 2.0 at 0.52 V, surpassing most previously reported carbon-based and/or noble-metal-based electrocatalysts (Fig. 2c and Table S6†). Comparatively, the S_0_RGO, S_1_RGO and S_20_RGO produce the H_2_O_2_ selectivities of 51–73%, 81–96% and 77–95% from 0.10 to 0.65 V, accompanied with the electron transfer numbers of 3.0–2.5, 2.4–2.0 and 2.5–2.1, respectively. The electron transfer numbers for S_*x*_RGO (*x* = 0, 1, 10, 20) obtained by the Koutecky–Levich (K–L) analysis are similar to those obtained by the RRDE method (Fig. S15 and S16†). It is noteworthy that, as shown in Fig. 2a, unlike the S_0_RGO catalyst, which exhibits a decreased ring current density at potentials below 0.3 V, the sulfur-doped catalysts, particularly S_10_RGO, demonstrate a slightly increased ring current density in the same potential range. This observation suggests that sulfur-doping can facilitate the formation of H_2_O_2_ in the low-potential region. Besides, S_10_RGO delivers an early onset potential of 0.773 V, surpassing those of S_1_RGO (0.762 V), S_20_RGO (0.767 V), and S_0_RGO (0.733 V). This early onset potential is superior to or comparable with most of the carbon-based catalysts reported hitherto.^15,44–46^ Regarding the kinetic current of H_2_O_2_ (*j*_k_), it is notable that S_10_RGO exhibits the highest value of 34.7 mA cm^−2^ at 0.50 V, vastly exceeding those of S_1_RGO (10.6 mA cm^−2^), S_20_RGO (32.9 mA cm^−2^) and S_0_RGO (6.6 mA cm^−2^) (Fig. 2d). However, the most active sample at a higher potential of 0.65 V is found to be S_20_RGO with a *j*_k_ value of 1.62 mA cm^−2^. Fig. 2e shows the S_*x*_RGO (*x* = 1, 10, 20) exhibit a smaller Tafel slope compared to that of S_0_RGO, indicating the doped sulfur content favoring ORR kinetics. Importantly, the mass activity of sulfur-doped catalysts, as depicted in Fig. S17,† surpasses that of S_0_RGO, with the highest value of 8.8 A g^−1^ observed for S_10_RGO at 0.75 V. This outperforms the performance of most reported carbon catalysts (Table S7†). The above results prove that incorporation of C–S and C–SO_*x*_ groups could greatly enhance the selectivity and activity of S_*x*_RGO (*x* = 1, 10, 20) towards 2e^−^ ORR, and the H_2_O_2_ selectivity and activity are dependent on the content of sulfur configurations (Fig. 2f and S18†). To further validate the catalytic trends induced by sulfur doping, two additional samples designated as S_5_RGO and S_15_RGO were prepared. Their surface elemental compositions, chemical states, and electrocatalytic ORR performances were compared with those of S_*x*_RGO (where *x* = 1, 10, 20) (Fig. S19†). The results confirmed the previously proposed structure–property correlation. The stability of S_10_RGO was assessed at a constant disk potential of 0.52 V (Fig. 2g), which shows that the H_2_O_2_ selectivity remained above 95% throughout 8 h of continuous electrolysis, showing commendable stability in an alkaline solution.

In addition, these sulfur-doped RGO samples exhibit remarkable selectivity towards H_2_O_2_ in a neutral solution (0.1 M Na_2_SO_4_) (Fig. S20†). As shown in Fig. S21,† the S_10_RGO displays significantly higher ring current and smaller disk current density with respect to the S_0_RGO, showing excellent H_2_O_2_ generation performance with a selectivity of 89% and an electron transfer number of 2.2 at 0.4 V. It has been demonstrated that the H_2_O_2_ reduction reaction (PRR) on the catalyst is an important factor affecting the cumulative formation of H_2_O_2_, so the electrocatalytic PRR experiments were conducted in Ar-saturated 0.1 M KOH and 0.1 M Na_2_SO_4_ solutions containing 50 mM H_2_O_2_, respectively. As shown in Fig. 2h and S21c,† the S_10_RGO delivers the lowest reduction current density in both KOH (0.18 mA cm^−2^) at 0.10 V and Na_2_SO_4_ (0.16 mA cm^−2^) at 0 V, which should be another important parameter for its superior performance in H_2_O_2_ production.

### Electrosynthesis of H_2_O_2_

**Fig. 3:**
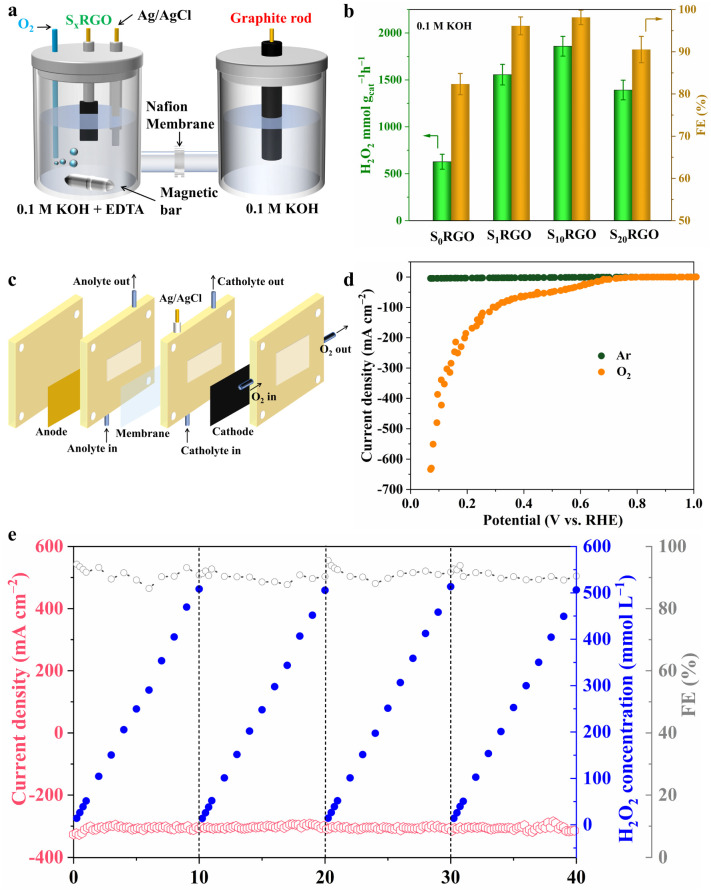
(a) Schematic illustration of the H-type cell for H_2_O_2_ production. (b) H_2_O_2_ production rate and FE of S_*x*_RGO in the H-type cell. (c) Schematic illustration of the flow cell setup for H_2_O_2_ production. (d) LSV curves of S_10_RGO tested under O_2_ and Ar atmospheres in the flow-cell setup, respectively. (e) Chronoamperometry test of S_10_RGO at 0.15 V for H_2_O_2_ production in the flow cell using 1.0 M KOH as the electrolyte and FE. The electrolyte was refreshed every 10 h during the test. Catalyst loading: 600 μg cm^−2^.

By casting S_10_RGO onto a hydrophobic carbon paper with a loading of 600 μg cm^−2^, the electrolysis experiment was conducted at 0.52 V using a custom-designed electrochemical H-type cell in O_2_-saturated 0.1 M KOH solution under 400 rpm stirring (Fig. 3a and S22†). The H_2_O_2_ production rates and Faraday efficiency (FE) over S_*x*_RGO were quantified by cerium sulfate (Ce(SO_4_)_2_) titration (Fig. S23†). As shown in Fig. 3b, the S_10_RGO displays a maximal H_2_O_2_ production rate (1853.85 ± 300 mmol g_cat_^−1^ h^−1^) and maximum FE (96.51 ± 3%) after 1 h with respect to the other three samples. Specifically, the S_10_RGO exhibits an almost linear increase in H_2_O_2_ yield over 60 min (Fig. S24†). As shown in Fig. S25,† a steady current density of ∼5 mA cm^−2^ could be maintained after 90 h without an obvious decline. It should be pointed out that as depicted in Fig. S26,† the catalytic effect of the carbon paper electrode can be disregarded since both the current and H_2_O_2_ generation predominantly originated from the S_10_RGO catalyst. Furthermore, a gas diffusion electrode and a three-phase flow cell reactor (Fig. 3c) were employed to enhance the H_2_O_2_ productivity of S_10_RGO by circumventing the issue of low O_2_ solubility in the aqueous electrolyte. The LSV curves were recorded in the flow-cell setup in 1.0 M KOH with manual 100% *iR* compensation. As depicted in Fig. 3d, the S_10_RGO demonstrates a significantly enhanced current density in an O_2_ atmosphere with respect to an Ar atmosphere in the flow-cell. The substantial disparity between the O_2_ and Ar atmospheres indicates the oxygen reduction ability of the cathode. Moreover, it is found that the polarisation curve of S_10_RGO seems to experience a kind of plateau at 0.5–0.3 V *vs.* RHE before a second wave kicks in, which is different from S_0_RGO (Fig. S27†). This unusual shape of the polarization curve shares similarities with the enhanced ring current density observed in LSV curves below 0.3 V (Fig. 2a). This phenomenon may stem from the oxidation of some of the C–S bonds to C–SO_*x*_ species during accumulation of H_2_O_2_ from 0.5 to 0.3 V, which would influence the interfacial interaction between the catalyst and H_2_O (Fig. S28†).^34^ This hypothesis was further proved by EIS measurements (Fig. S29 and S30†), which indicated that the mass transport kinetics was retarded in S_10_RGO in the potential range of 0.3–0 V, thereby creating favorable interfacial conditions for selective H_2_O_2_ synthesis through inhibiting the attack of H_2_O_2_ by active hydrogen.^47^ Surprisingly, the S_10_RGO attains a remarkable current density of up to 500 mA cm^−2^ at a low potential of merely 0.10 V, manifesting significant potential for industrial applications. During the bulk electrolysis at different potentials, a high current density ranging from 50 to 300 mA cm^−2^ can be consistently maintained without noticeable degradation for at least 40 h, respectively (Fig. S31–S33†). Moreover, at an industrial current density of 300 mA cm^−2^, the S_10_RGO exhibited an average FE% of approximately 90.5% and achieved a high production rate of 9.33 ± 0.19 mol g^−1^ h^−1^, respectively. Compared to literature catalysts (Table 1), S_10_RGO's wide operational window and balanced performance metrics underscore its potential for industrial-scale applications. The concentration of the accumulated H_2_O_2_ increased almost linearly and reached 508.2 mmol L^−1^ after about 10 h of electrolysis, which could be stably operated for 4 cycles and produced 0.4 L of 508.2 mmol per L H_2_O_2_ solution (Fig. 3e). The long-term accumulation of H_2_O_2_ at a potential of 0.15 V over a period of 29 h was executed in a flow cell containing 100 mL of alkaline electrolyte, as illustrated in Fig. S34;† throughout this interval, the cumulative amount of H_2_O_2_ reached a significant 857.5 mmol L^−1^ (equivalent to 3.0 wt%), which is deemed adequate for the production of medical-grade disinfectants.^55^ These results emphasize the capability of S_10_RGO for continuous and stable production of H_2_O_2_, suggesting its significant potential as a feasible candidate for large-scale H_2_O_2_ electrosynthesis in industrial applications.

**Table 1:** Performance parameters of S_10_RGO and various reported catalysts in alkaline media

Catalyst	Selectivity (%)	Stability	Production (mol g_cat_^−1^ h^−1^)	FE (%)
S_10_RGO	90–98.9% @ 0.1–0.65 V	40 h @ 300 mA cm^−2^	9.33 ± 0.19	∼90.5
		50 h @ 120 mA cm^−2^	6.74 ± 0.18	∼91
		50 h @ 50 mA cm^−2^	4.28 ± 0.15	∼91.3
This work				
N-mFLG-8 (ref. 16)	95–98.5% @ 0.3–0.7 V	50 h @ 20 mA cm^−2^	9.66	∼100
OCNS_900_ (ref. 48)	90–91% @ 0.55–0.75 V	11 h @ 50 mA cm^−2^	0.77	—
CNB-ZIL-8 (ref. 49)	80–85% @ 0.2–0.6 V	9 h @ 40 mA cm^−2^	1.787	∼80
Thiophene-S^29^	90–93% @ 0.5–0.75 V	8 h @ 20 mA cm^−2^	3.46	∼92.8
HPCS-S^25^	70% @ 0.3 V	—	183.99 (H-cell)	—
S-DNC^26^	90% @ 0.78 V	—	4.05 (H-cell)	∼100
S-Nv-C_3_N_4_ (ref. 27)	95% @ 0.35 V	24 h @ 200 mA cm^−2^	4.52 (H-cell)	∼80
S-mC-0.375 (ref. 30)	92–99% @ 0.2–0.7 V	24 h @ 185 mA cm^−2^	25	∼95
B–C^15^	90.5% @ 0.65 V	30 h @ 200 mA cm^−2^	—	85–90
Co_1_–NG(O)^8^	82% @ 0.1 V	110 h @ 5 mA cm^−2^	—	∼93
Pb–SA/OSC^50^	90–94% @ 0.3–0.7 V	2 h @ 400 mA cm^−2^	0.69	∼92.7
Co HSACs^51^	95% @ 0.5–0.75 V	25 h @ 300 mA cm^−2^	—	∼90
NBO–G/CNTs^52^	80–100% @ 0.2–0.7 V	12 h @ 50 mA cm^−2^	0.709	∼80
Co–N_5_–O–C SACs^53^	80–85% @ 0.3–0.75 V	24 h @ 100 mA cm^−2^	5.92	∼80
Sb-NSCF^54^	90–97.2% @ 0.4–0.7 V	75 h @ 50 mA cm^−2^	7.46	∼80

### Understanding the underlying mechanism

**Fig. 4:**
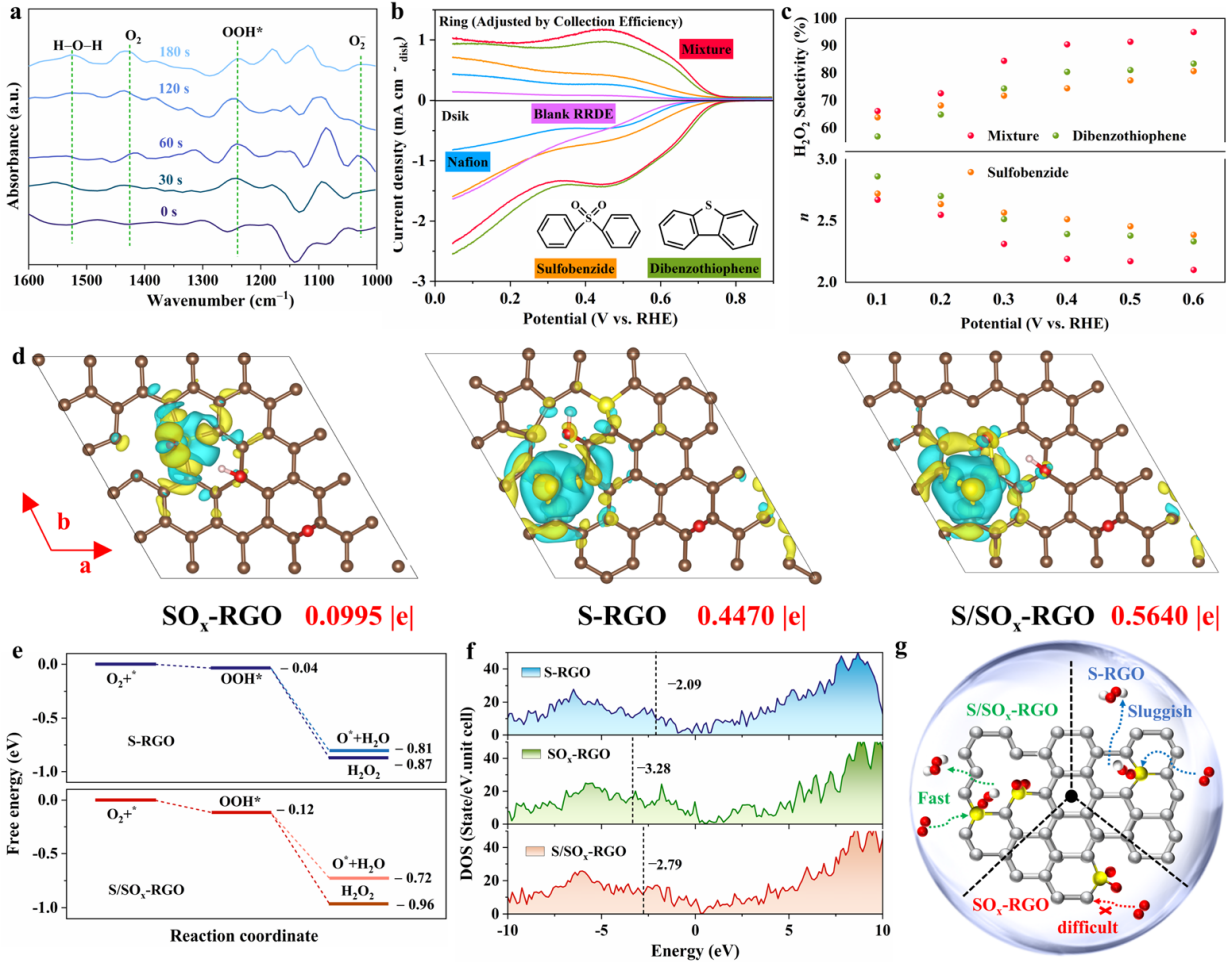
(a) ATR-SEIRAS spectra recorded on S_10_RGO at different time intervals in O_2_-saturated 0.10 M Na_2_SO_4_. (b) LSV curves and (c) H_2_O_2_ selectivity of standalone molecules and their mixture. Catalyst loading: 143 μg cm^−2^. (d) Differential charge densities of SO_*x*_-RGO, S-RGO and S/SO_*x*_-RGO. Yellow and cyan isosurfaces show the electron gain and electron loss, respectively. (e) The free energy variations for the S-RGO and S/SO_*x*_-RGO during 2e^−^ and 4e^−^ ORR. (f) The density of states (DOS) for SO_*x*_-RGO, S-RGO and S/SO_*x*_-RGO. The dashed lines are Fermi levels. (g) Schematic diagram of two-electron pathways on S_*x*_RGO with different S configurations.

To detect the key adsorbed oxygen intermediate on the S_10_RGO during electrocatalytic H_2_O_2_ synthesis, *in situ* attenuated total reflectance surface-enhanced infrared absorption spectra (ATR-SEIRAS) were recorded at different time intervals and potentials in the O_2_-saturated 0.10 M Na_2_SO_4_ and 0.10 M KOH solution (Fig. 4a and S35†). Notably, two new absorption peaks at ∼1240 cm^−1^ and 1420 cm^−1^ emerged and increased over time, which can be assigned to the O–O stretching vibration of OOH* and O–O stretching mode of adsorbed molecular oxygen (O_2,ad_), respectively.^56,57^ Therefore, the ATR-SEIRAS results supported the OOH*-mediated 2e^−^ ORR pathway.

Secondly, the sulfur-containing molecule investigations were designed and conducted to uncover the effects of C–S and sulfur oxide species in the 2e^−^ ORR.^58^ Small organic molecules including dibenzothiophene (DS), sulfobenzide (SO) and their mixture were selected as heterogeneous catalysts. Their electrocatalytic ORR performances were evaluated *via* a similar process. Meanwhile, comparison experiments using a blank RRDE or a 5% Nafion solution modified RRDE were performed. It is important to highlight that, owing to the distinct differences in interaction modes, interface environments, and accessibility of active sites between sulfur-containing molecule-loaded heterogeneous catalysts and sulfur-doped carbon solid catalysts, the electrocatalytic performance of molecular catalysts is significantly inferior to that of sulfur-doped carbon catalysts in terms of both activity and selectivity. Interestingly, as shown in Fig. 4b and c, the mixture of DS and SO exhibited superior performance in terms of H_2_O_2_ selectivity, kinetic current of H_2_O_2_ and Tafel slope as compared to other single catalysts (Fig. S36†). This phenomenon further supports the above research results that the C–S and C–SO_2_ groups show a synergistic effect in driving 2e^−^ ORR.

Finally, to reveal the role of different sulfur configurations in electrocatalytic 2e^−^ ORR, DFT calculations were employed to simulate the charge density, H_2_O_2_ formation pathway and Fermi levels of three typical catalysts (S-RGO, SO_*x*_-RGO, and S/SO_*x*_-RGO) containing different sulfur configurations (Fig. S37†). The 2e^−^ ORR pathway exclusively involves the intermediate OOH*, while further reducing OOH* generates intermediates (O* and OH*) that proceed *via* the 4e^−^ ORR pathway. It has been reported that the formation of the OOH* intermediate is the rate-limiting step for achieving selectivity towards H_2_O_2_.^59^ As seen in Fig. 4e and S38,† the first step (O_2_* → OOH*) over the RGO and SO_*x*_-RGO is shown to be endergonic, while the S-RGO and S/SO_*x*_-RGO exhibit exergonic behavior. In other words, the challenging limiting step on the RGO and SO_*x*_-RGO surfaces becomes facile on the S-RGO and S/SO_*x*_-RGO surfaces. Compared to S-RGO, S/SO_*x*_-RGO further reduces the energy barrier of the limiting step O_2_* → OOH*. Besides, the free energy drop for H_2_O_2_ formation (OOH* → H_2_O_2_) over S/SO_*x*_-RGO (−0.84 eV) is slightly larger than that over S-RGO (−0.83 eV), indicating a favorable propensity for H_2_O_2_ production over S/SO_*x*_-RGO. Additionally, the formation energy of OOH* to O* is higher over S/SO_*x*_-RGO (−0.60 eV) compared to that over S-RGO (−0.77 eV), indicating its reduced susceptibility to the 4e^−^ process. Therefore, the ORR activity trend follows the order: RGO < SO_*x*_-RGO < S-RGO < S/SO_*x*_-RGO, and the S/SO_*x*_-RGO serves as a highly favorable catalyst for 2e^−^ ORR, which agrees with the S_*x*_RGO experimental and standalone molecule investigation results.

The effects of the functional groups on the formation of OOH* and H_2_O_2_ can be further explained by the charge density and Fermi levels. As depicted in Fig. 4d, the charge depletion is shown as the region of C atoms near sulfur oxide on the SO_*x*_-RGO (0.0995 e^−^), whereas in the cases of S-RGO and S/SO_*x*_-RGO, the charge state of the S atom becomes more positive by 0.5640 e^−^ and 0.4470 e^−^, respectively, in qualitative agreement with that of the previous report.^60^ This suggests that the C–S group is primarily utilized to increase the charge state of the S atom, while the electronic charge is depleted towards the neighboring C atoms, specifically facilitating the originally difficult rate-limiting step (O_2_* → OOH*) over RGO. Meanwhile, when both C–S and C–SO_*x*_ groups coexist in the RGO, the electron-poor sulfur center serves as O_2_ adsorption sites on the graphitic carbon matrix, which facilitates the adsorption of O_2_ molecules and reduces the barrier to O_2_* → OOH*. On the other hand, as shown in Fig. 4f, the presence of the C–SO_*x*_ group can lower the Fermi level of the catalyst compared to the C–S group. This decrease in Fermi level weakens the adsorption of the OOH* intermediate since it becomes more challenging to donate electrons from the catalyst, which facilitates the release of OOH* and effectively prevents the 2e^−^ + 2e^−^ reaction mechanism.^8^ As a consequence, the C–S and C–SO_*x*_ groups play distinct yet complementary roles in the electrocatalytic 2e^−^ ORR. The suitable sulfur configuration and proportion gives rise to favorable formation and optimal adsorption strength of the *OOH intermediate, further achieving exceptional 2e^−^ ORR selectivity for the sulfur-doped carbon material (Fig. 4g).

## 4 Conclusions

In this work, the C–S and C–SO_*x*_ active centers in sulfur-doped RGO materials (S_*x*_RGO) for 2e^−^ ORR were successfully constructed and identified on the basis of an experimental investigation and theoretical calculations. By temperature-programmed annealing and adjusting the mass ratio of precursor materials, the sulfur configurations on the S_*x*_RGO catalysts can be tuned and optimized. The C–S and C–SO_*x*_ groups have complementary effects during 2e^−^ ORR, in which the C–S group greatly drives the rate-limiting step (O_2_* → OOH*) by increasing the charge state of the S atom and promoting adsorption of O_2_, while C–SO_*x*_ species modulate the ORR pathway through both electronic and interfacial effects, weakening OOH* adsorption and tuning proton transfer dynamics. Finally, the optimized sulfur configurations on the S_10_RGO catalyst exhibit excellent 2e^−^ ORR performance in alkaline media, with a maximum selectivity of 98.9% for electrochemical H_2_O_2_ synthesis and selectivity exceeding 90% across a wide voltage range of 0.1–0.65 V. The S_10_RGO exhibits a current density of up to 500 mA cm^−2^ for H_2_O_2_ production in a flow cell device, achieving a production rate of 9.33 ± 0.19 mol g_cat_^−1^ h^−1^ and maintaining a stable FE of 90.5% during the ORR at a current density of 300 mA cm^−2^ for 40 h. Our findings should contribute to developing efficient carbon-based catalysts for electrocatalytic production of H_2_O_2_ in practical industrial applications.

## Data availability

The data that support the findings of this study are available from the corresponding authors upon reasonable request.

## Author contributions

Sifan Li: investigation, data curation, formal analysis, visualization, writing – original draft. Shiwen Du: investigation, data curation, software, writing – original draft, writing – review & editing. Jiansheng Li: conceptualization, methodology, validation, writing – review & editing, supervision, funding acquisition. Wenjun Fan: formal analysis, methodology. Yang Yang: formal analysis, methodology. Peng Zhao: data curation, visualization. Haotian Zhu: software, validation. Wansheng You: methodology, visualization. Xiaojing Sang: formal analysis, methodology, validation, writing – review & editing. Fuxiang Zhang: conceptualization, methodology, validation, writing – review & editing.

## Conflicts of interest

There are no conflicts to declare.

## Supplementary Material

SC-OLF-D5SC03069B-s001
