# Supplementary material for: Metal-free sulfur-doped reduced graphene oxide electrocatalysts for promising production of hydrogen peroxide: construction and identification of active sites
Source: Chem Sci. 2025 Jun 2;16(26):12178–88. doi: 10.1039/d5sc03069b (PMC12146891; doi:10.1039/d5sc03069b)
Supplement: SC-016-D5SC03069B-s001 [file SC-016-D5SC03069B-s001.pdf]

Supplementary Information (SI) for Chemical Science.
This journal is © The Royal Society of Chemistry 2025

Supporting Information

Metal-free sulfur-doped reduced graphene oxide electrocatalysts for promising production of hydrogen peroxide: construction and identification of active sites

Sifan Li, †[ad] Shiwen Du, †[bc] Jiansheng Li, *[ab] Wenjun Fan, [b] Yang Yang, [b] Peng Zhao, [a] Haotian Zhu, [a] Wansheng You, [a] Xiaojing Sang *[a] and Fuxiang Zhang*[b]

[a]School of Chemistry and Chemical Engineering, Liaoning Normal University, Dalian 116029, Liaoning, China

[b]State Key Laboratory of Catalysis, Dalian Institute of Chemical Physics, Chinese Academy of Sciences, Dalian, 116023, Liaoning, China

[c]School of Physics and Materials Engineering, Dalian Minzu University, Dalian, 116023, Liaoning, China

[d]Department of Biochemical Engineering, Chaoyang Normal University, Chaoyang 122000, Liaoning, China

†These authors contributed equally to this work.

Materials

GO was purchased from Jicang Nano Technology Co., Ltd. Sulfur sublimated (≧ 99.5%) was purchased from Xilong Science Co., Ltd. Here, sublimed sulfur was selected as the sulfur precursor instead of regular sulfur, in consideration of the superior purity and metastable structure of sublimed sulfur compared to regular sulfur, both of which are critical for optimizing the sulfur-doped RGO materials. To be specific, sublimed sulfur (≥99.5% purity) is purified via sublimation-condensation, removing organic residues and metal impurities that persist in regular sulfur (95–98% purity). This minimizes side reactions during composite formation, as impurities in regular sulfur can catalyze polysulfide shuttling.[1] Furthermore, sublimed sulfur forms metastable *β*-S or amorphous phases with smaller crystallites (1–10 μm) and higher surface area (5–10 $m^2\ g^{-1}$), enabling homogeneous dispersion in GO. In contrast, regular sulfur exists as aggregated *α*-S crystals (>50 μm, <1 $m^2\ g^{-1}$), which hinder interfacial bonding. These structural advantages directly contribute to improved electrochemical performance.[2] Hydrochloric acid (35%−37%), acetone (99.5%) were purchased from Kemio Co., Ltd. Cerium sulfate ($Ce(SO_4)_2$), sodium sulfate (Na_2SO_4), dibenzothiophene ($C_{14}H_8S$), sulfobenzide ($C_{12}H_{10}O_2S$) were purchased from Aladdin Co., Ltd. Nafion solution (5 wt%) was purchased from Dupont Co., Ltd. All the chemicals were directly used without further processing.

Characterization

Fourier Transform Infrared Spectroscopy (FTIR) was recorded on a Bruker AXS TENSOR-27 FTIR spectrometer. Samples were pelletized with KBr and the data were collected over in the range of 4000–400 cm^{-1}. X-ray powder diffraction (XRD) data were collected on a Bruker AXS D8 Advance diffractometer using Cu K*α* radiation (λ = 1.5418 Å). X-ray Photoelectron Spectroscopy (XPS) analysis was operated on a VG ESCALAB MKII spectrometer using Mg Kα (1253.6 eV) achromatic X-ray source. The spectra were calibrated with reference to the amorphous C1s peak of 284.6 eV. The scanning electron microscopy (SEM) was carried out on a JEOL ZXM6360-LV. The morphology of the nanostructured materials was characterized by an FEI Technai G2 F20 transmission electron microscope (TEM). Raman scattering spectroscopy was performed on a Horiba Scientific LabRam HR Evolution, equipped with a 532 nm Argon

laser. Ultraviolet-visible (UV-Vis) absorption spectra were obtained using a UV-Vis spectrometer (UV-1700PC, Macy). Thermogravimetric (TGA) analysis was performed on a Pyris Diamond TG-DTA thermal analyzer at a heating rate of 10 °C min^{-1} in flowing N_2.

Rotation ring disk electrode (RRDE) test

The electrochemical experiments were performed on the CHI 760E electrochemical workstation utilizing a three-electrode system. An RRDE (Taizhou Keruite Analytical Instrument, Co., Ltd, disk area: 0.1256 cm^2) with a Pt ring (ring area: 0.1664 cm^2) was used as the working electrode, a graphite rod as the counter electrode and an Ag/AgCl electrode as the reference electrode, respectively. To prepare the catalyst ink, 3.0 mg of the obtained catalysts were mixed in 1 mL of a solution containing 980 µL of deionized water/ethanol mixing solution ($V_{water}/V_{ethanol}$ = 5:1) and 20 µL of 5 wt% Nafion solution. The mixture was then subjected to ultrasonic treatment for 60 min to form homogeneous inks. Before measurement, the RRDE was polished with 0.30, 0.10 and 0.05 µm alumina powders (Chenhua) and then cleaned with deionized water. Subsequently, 6 µL (a loading of roughly 0.143 mg cm^{-2}) of the catalyst ink was drop-casted onto a disk electrode of the RRDE tip spinning at an initial rate of 150 rpm and advanced to 300 rpm to achieve uniform electrode coverage. Two electrolytes with pH ~13 (0.1 M KOH) and ~7 (0.1 M Na_2SO_4) were used at room temperature. All potentials measured against an Ag/AgCl electrode were converted to the reversible hydrogen electrode (RHE).

Prior to ORR measurements, the electrolyte was purged with O_2 gas for at least 15 min to achieve a saturated concentration of dissolved O_2 gas. Throughout the measurements, an O_2 gas blanket was maintained over the electrolyte surface to ensure stable dissolved O_2 gas concentration. The disk was first conditioned in O_2-saturated electrolyte by performing cyclic voltammetry (CV) between 0 V and 0.90 V at a scan rate of 100 mV s^{-1} and a rotation speed of 1600 rpm for 40 cycles, meanwhile holding the platinum ring at 1.30 V. Subsequently, the platinum ring was cleaned in O_2-saturated electrolyte by performing CV between 0 V and 1.20 V at a scan rate of 100 mV s^{-1} and a rotation speed of 1600 rpm for 40 cycles, meanwhile holding the disk

potential constant at 0.80 V. The H_2O_2 productivity and selectivity were determined through linear sweep voltammetry (LSV) under O_2-saturated conditions, with a scan rate of 10 mV s^{-1} at 1600 rpm, within the potential range of 0 V to 0.90 V meanwhile maintaining the platinum ring electrode potential at 1.30 V. The collection efficiency (N) was determined to be 0.43 by the redox reaction of $[Fe(CN)_6]^{4-}/[Fe(CN)_6]^{3-}$ according to the reported procedures.[3] Accordingly, the H_2O_2 selectivity was calculated using the equation:

$$H_2O_2\% = 200\times\frac{\left|\frac{j_{\text{ring}}}{N}\right|}{|j_{\text{disk}}|+\frac{j_{\text{ring}}}{N}} \tag{1}$$

The electron transfer number (n) at the disk electrode during ORR process was calculated using the equation:

$$n = 200\times\frac{/j_{\text{disk}}/}{/j_{\text{disk}}/+\frac{/j_{\text{ring}}/}{N}} \tag{2}$$

where j_{disk} and j_{ring} are the disk and the ring current, respectively, and N is the collection efficiency.

The electron transfer number (n) can also be determined using the Koutecky-Levich (K-L) method, which characterizes the behavior of the disk current density:

$$\frac{1}{j_{\text{disk}}} = \frac{1}{j_k}+\frac{1}{j_L} \tag{3}$$

$$j_L = 0.62\times n\times F\times D^{2/3}\times v^{-1/6}\times C\times\omega^{1/2} \tag{4}$$

$$j_{\text{disk}}^{-1} = \frac{1}{j_k}\times\frac{1}{0.62\times n\times F\times D^{2/3}\times v^{-1/6}}\times\omega^{-1/2} \tag{5}$$

Where j_k and j_L are the kinetic and diffusion-limited current density, respectively, F, D, v, C_0 and ω indicate the Faraday constant (96485 C mol^{-1}), diffusion coefficient of O_2 in the electrolyte at 298 K (1.85×10^{-5} cm^2 s^{-1}), kinematic viscosity of the electrolyte (0.89×10^{-2} cm^2 s^{-1}), concentration of O_2 in the bulk electrolyte (1.21×10^{-6} mol cm^{-3}), and electrode rotation speed (rad s^{-1}) of the disk electrode.

The Tafel plots were generated according to the equation:

$$\eta = b\log j_k + a \tag{6}$$

Where η, a, j_k and b are the applied potential, constant, kinetic current for H_2O_2 production and Tafel slope, respectively. The j_k were generated according to the equation:

$$\frac{1}{j} = \frac{1}{j_k} + \frac{1}{j_l} \tag{7}$$

Where j and j_l are the measured current density and diffusion-limited current density for H_2O_2 production, respectively. The j value was calculated by dividing the ring current by the disk electrode area and the collection efficiency (N). The j_l value was obtained from the maximum value in the j plot measured across the entire investigated potential range.

The double-layer capacitance was determined from cyclic voltammograms in the non-faradaic region at different scan rates (20, 40, 60, 80 and 100 mV s^{-1}). Chronoamperometry test was performed at disk electrode potential of 0.52 V in O_2-saturated 0.1 M KOH electrolyte with an RRDE rotating speed of 1600 rpm. The Pt ring electrode was cleaned by rapid CV scanning from 0 V to −0.3 V and the electrolyte was refreshed every 2 h during the continuous operation. The measured potentials using a three-electrode setup of RRDE have no *iR* compensation.

In-situ ATR-SEIRAS measurements

In situ attenuated total reflectance surface-enhanced infrared absorption spectroscopy (ATR-SEIRAS) was conducted using a Nicolet iS50 FTIR spectrometer equipped with an MCT detector, which was cooled by liquid nitrogen during the testing process. The electrochemical measurement was performed in a custom-made three-electrode electrochemical single cell, where the reference and counter electrodes were Ag/AgCl and graphite rod, respectively. A semi-cylindrical prism with a diameter of 20 mm, coated with a thin layer of Au, was used as the working electrode after loading catalysts. In situ FTIR spectra were acquired by stepwise varying the potential from 0.9 V to 0 V in O_2-saturated 0.1 M Na_2SO_4, with a spectral resolution of 4 cm^{-1}. The spectrum obtained at open circuit voltage was used for background subtraction.

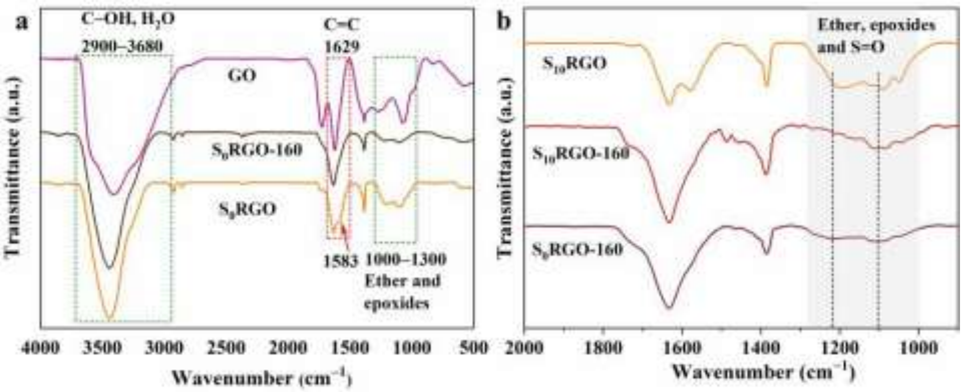

Fig. S1 FTIR spectra of GO, S_0RGO-160, S_{10}RGO-160, S_0RGO and S_{10}RGO.

The FTIR spectrum of S_0RGO shows a peak at 1629 cm^{-1} corresponding to the C=C stretching vibration in aromatic rings, which exhibits an extra shoulder peak at 1583 cm^{-1} compared to that of GO and S_0RGO-160.[4,5] The observation suggests that the formation of defects in RGO takes place with the temperature raising from 160 to 500 °C, which could induce the adsorption and incorporation of sulfur, resulting in the formation of C−S bond. Additionally, compared to S_0RGO-160, the peak corresponding to the S=O stretching vibration is observed at 1000−1300 cm^{-1} for S_{10}RGO-160, exhibiting overlapping peaks with ether and epoxides. This suggests that sulfur oxide species has been generated during the initial ramping step at 160 °C.

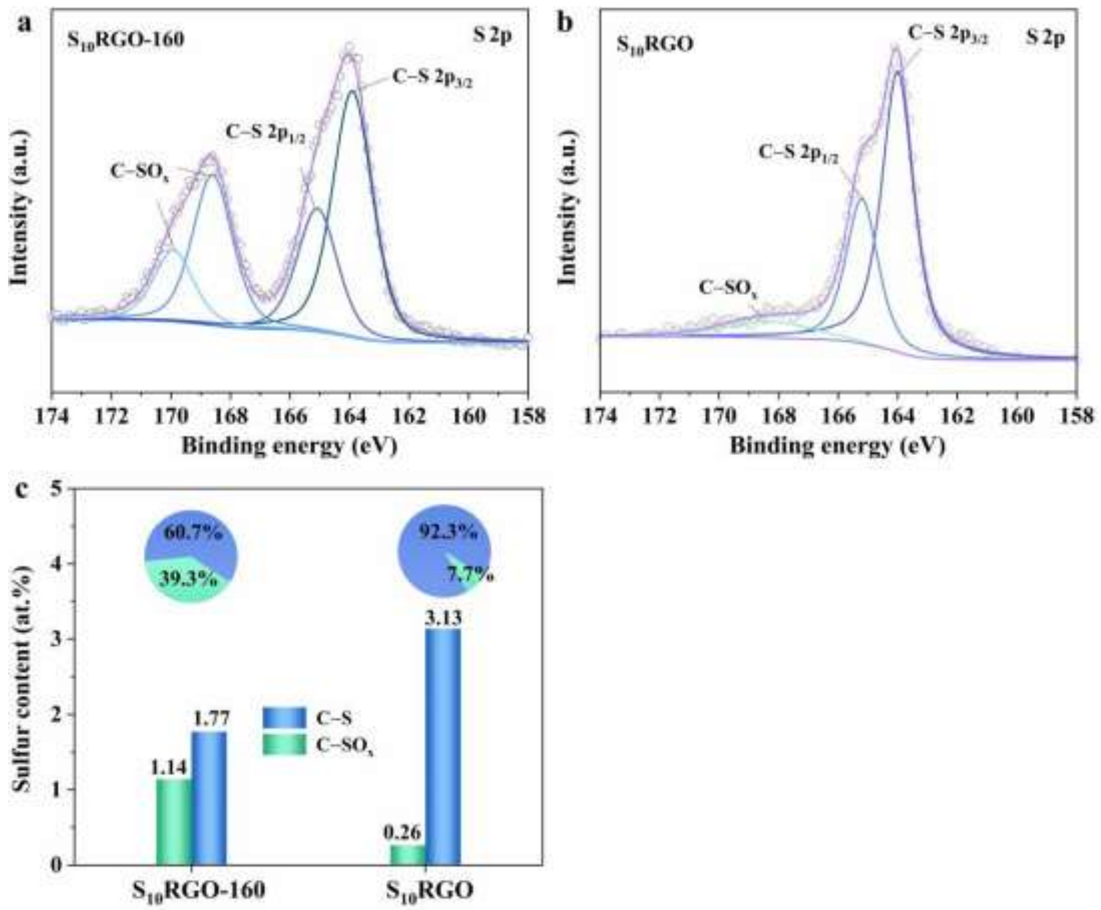

Fig. S2 High-resolution S 2p XPS spectra of (a) S_{10}RGO-160 and (b) S_{10}RGO. (c) Proportional content of each sulfur configuration (inset is sector diagram).

The high-resolution S 2p spectra of S_{10}RGO-160 and S_{10}RGO can be deconvoluted into S $2p_{3/2}$ (165.2 ± 0.1 eV), S $2p_{1/2}$ (164 ± 0.1 eV) of C–S group and C–SO_x group (167.5–170 eV).[6,7] It is found that the proportional content of sulfur oxide species in S_{10}RGO-160 is evidently higher than that in S_{10}RGO, while the proportional content of C–S group greatly increases in S_{10}RGO. This result infers that the sulfur oxide species are mainly formed during the initial ramping step at 160 °C and the second annealing step at 500 °C facilitates the formation of C–S groups. The $2e^-$ ORR performances of S_{10}RGO-160 are discussed below.

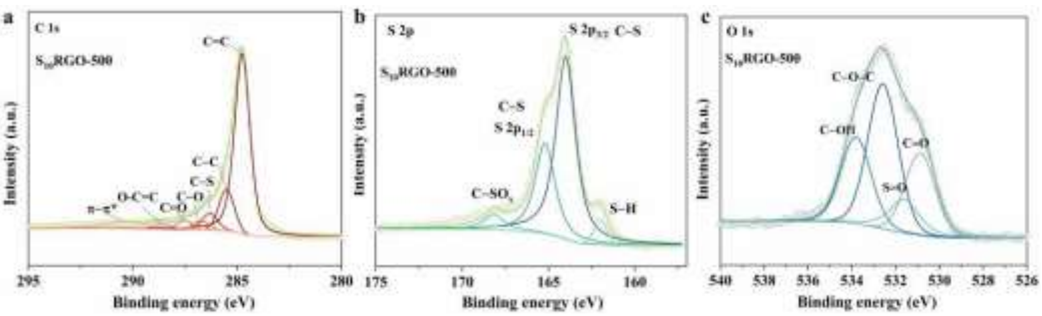

Fig. S3 High-resolution XPS spectra of S_{10}RGO-500. (a) C 1s, (b) S 2p, (c) O 1s.

The high-resolution C 1s spectra of S_{10}RGO-500 can be deconvoluted into distinct seven peaks, including the *sp²* C=C (284.7 eV), *sp³* C−C (285.5 eV), C−S (286.2 eV) C−O (286.7 eV), C=O (287.5 eV), O−C=O (288.8 eV) and π–π* shakeup (291.0 eV) features.[8] The high-resolution S 2p spectra can be deconvoluted into four peaks. These peaks are assigned to the following sulfur configurations: S–H (162.0 eV), S $2p_{3/2}$ (164 ± 0.1 eV), S $2p_{1/2}$ (165.2 ± 0.1 eV) corresponding to C–S, and C–SO_x (167.5–170 eV).[9] The high-resolution O 1s spectra can be deconvoluted into C−OH (534.0 eV), O−C−O (533.4 eV), S=O (531.8 eV) and C=O (530.8 eV) peaks.[9] The $2e^-$ ORR performances of S_{10}RGO-500 are discussed below.

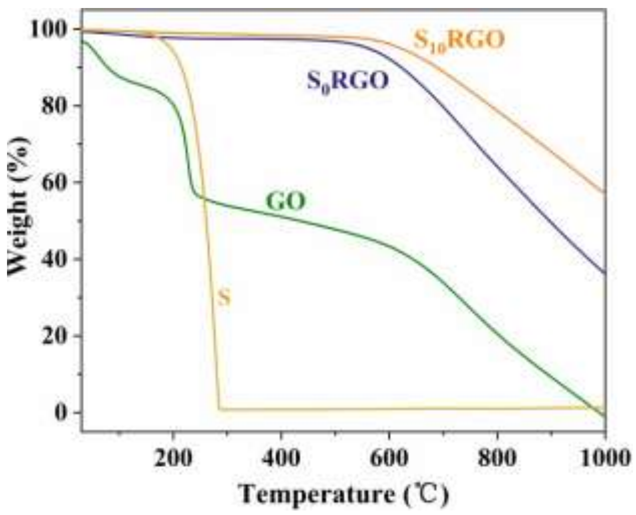

Fig. S4 TGA curves of sulfur powder, GO, S_0RGO and $S_{10}RGO$.

The thermogravimetric (TGA) curve also confirms the absence of crystal sulfur inclusion. In the nitrogen-protected thermogravimetric test, the temperature is gradually increased to 1000 °C at a constant rate of 10 °C min^{-1}. As depicted in Fig. S4, sublimed sulfur starts to melt and sublime around 150 °C with gradual loss, while no mass loss is observed for $S_{10}RGO$ at this temperature. This observation indicates that there is no crystal sulfur in $S_{10}RGO$. Simultaneously, it can be observed that the incorporation of sulfur enhances the thermal stability of the material.

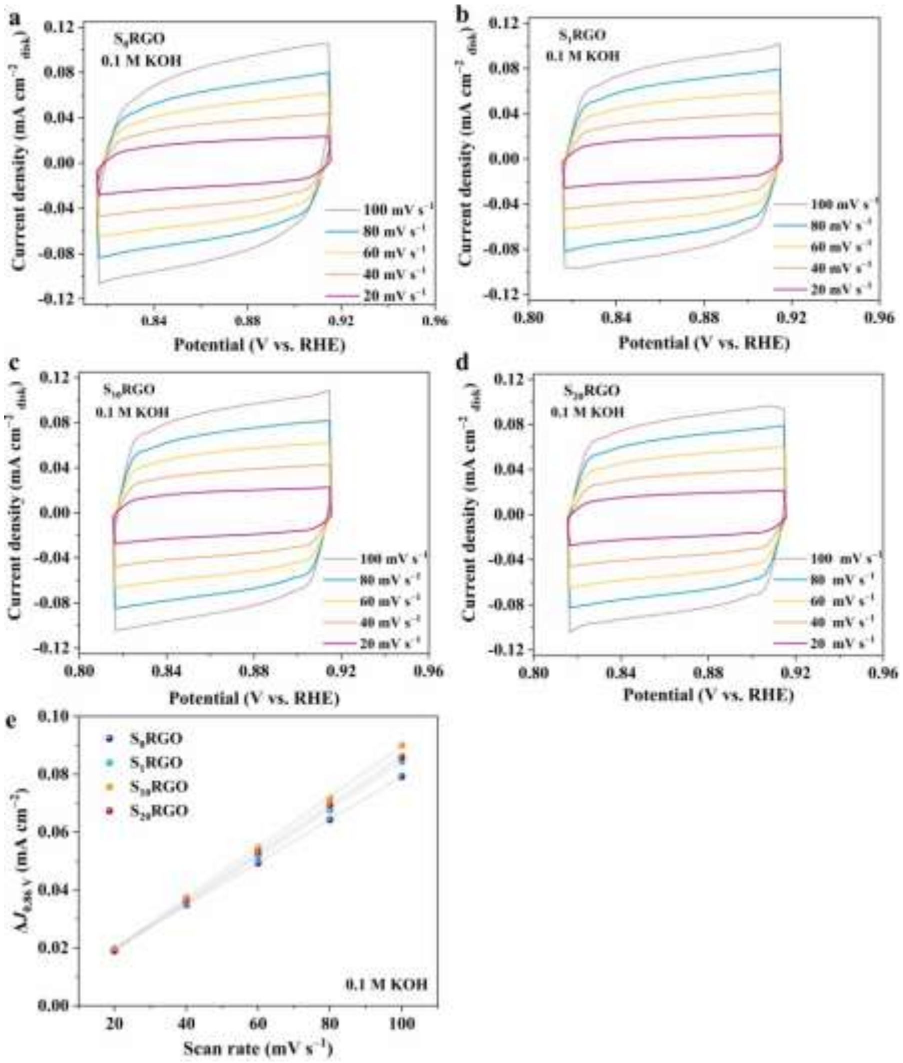

Fig. S5 (a–d) CV curves at non-faradaic potentials of S_xRGO (x= 0, 1, 10, 20) with scan rates from 20 to 100 mV s^{-1} and (e) the capacitive current at 0.86 V (vs. RHE) as a function of scan rate. Catalyst loading: 143 µg cm^{-2}.

The electrochemical active specific surface areas (ECSAs) of S_xRGO (x = 0, 1, 10, 20) were analyzed by recording the CV curves in the non-faradaic regions with results given in Fig. S5, based on which the values of double-layer capacitance (C_{dl}) and ECSA were derived and presented in Table S2. It is found that the S_xRGO (x = 1, 10, 20) show much higher ECSAs with respect to the S_0RGO, exposing much more active sites. Meanwhile, the S_xRGO (x = 1, 10, 20) samples exhibit similar ECSAs, indicating their analogous surface roughness.

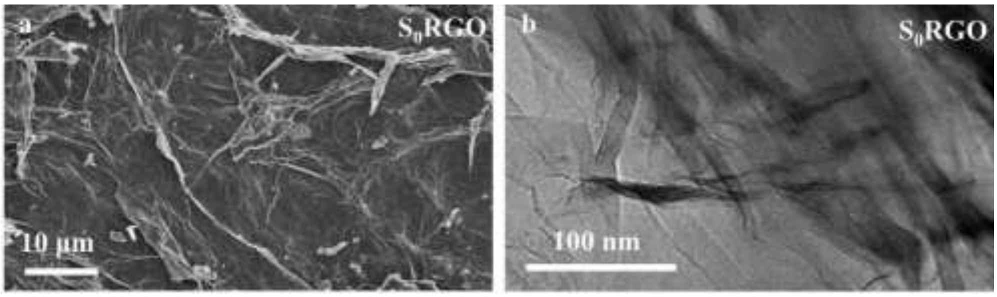

Fig. S6 (a) SEM and (b) TEM images of S_0RGO.

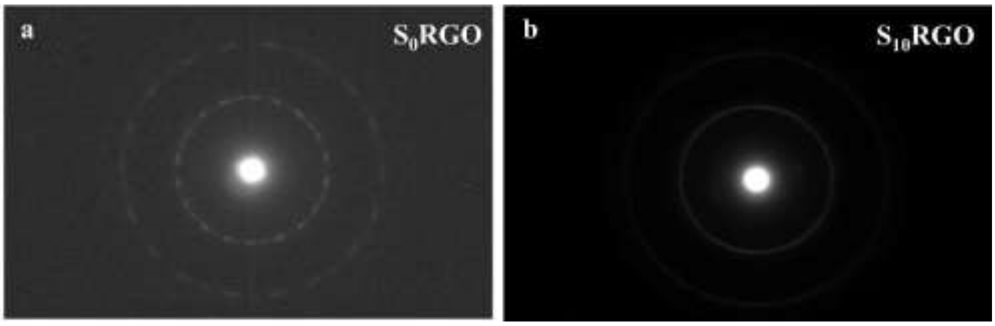

Fig. S7 SAED images of (a) S_0RGO and (b) $S_{10}RGO$.

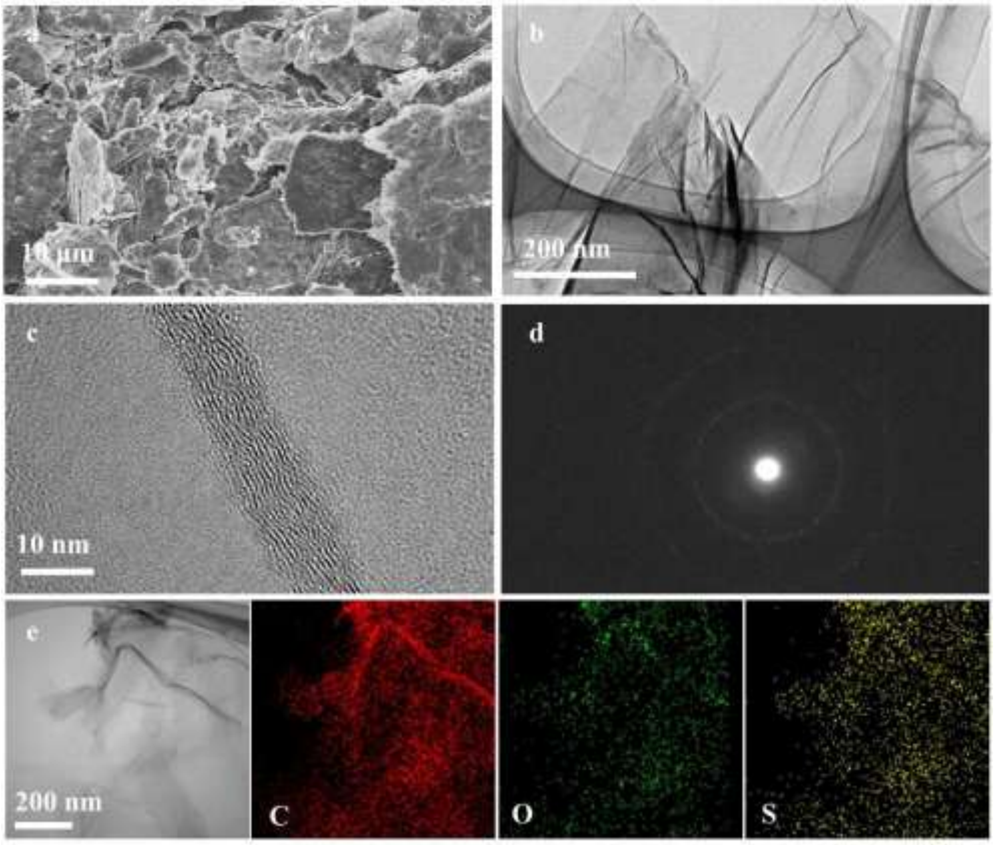

Fig. S8 The analysis of S_1RGO. (a) SEM image, (b) TEM image, (c) HR-TEM image, (d) SAED image and (e) element mapping.

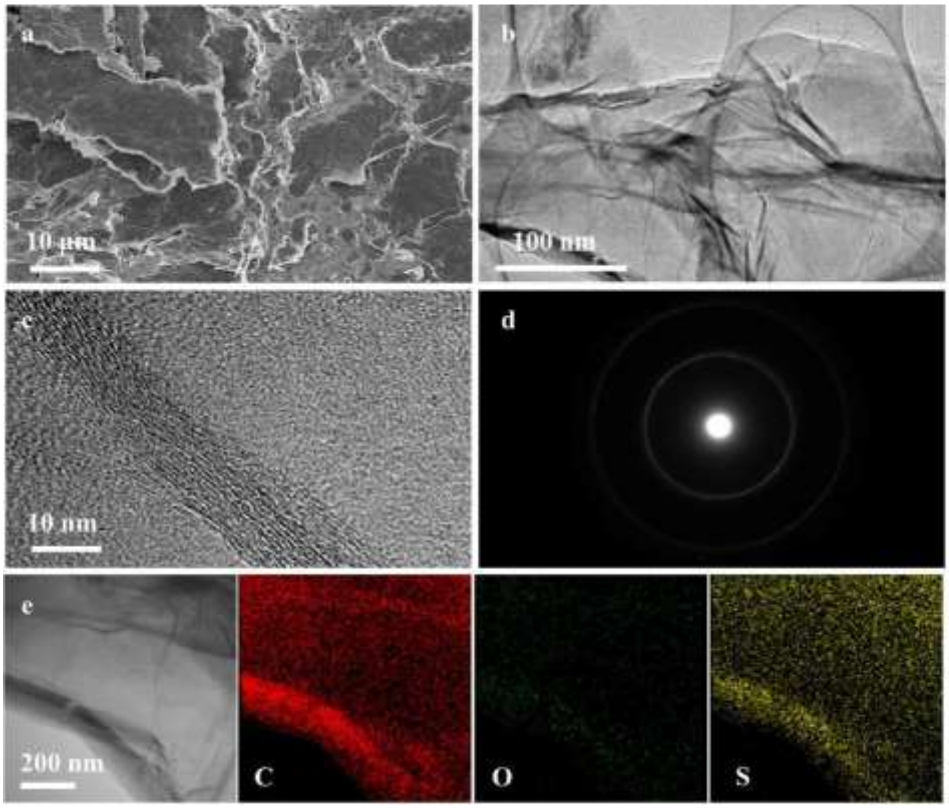

Fig. S9 The analysis of $S_{20}RGO$. (a) SEM image, (b) TEM image, (c) HR-TEM images, (d) SAED image and (e) element mapping.

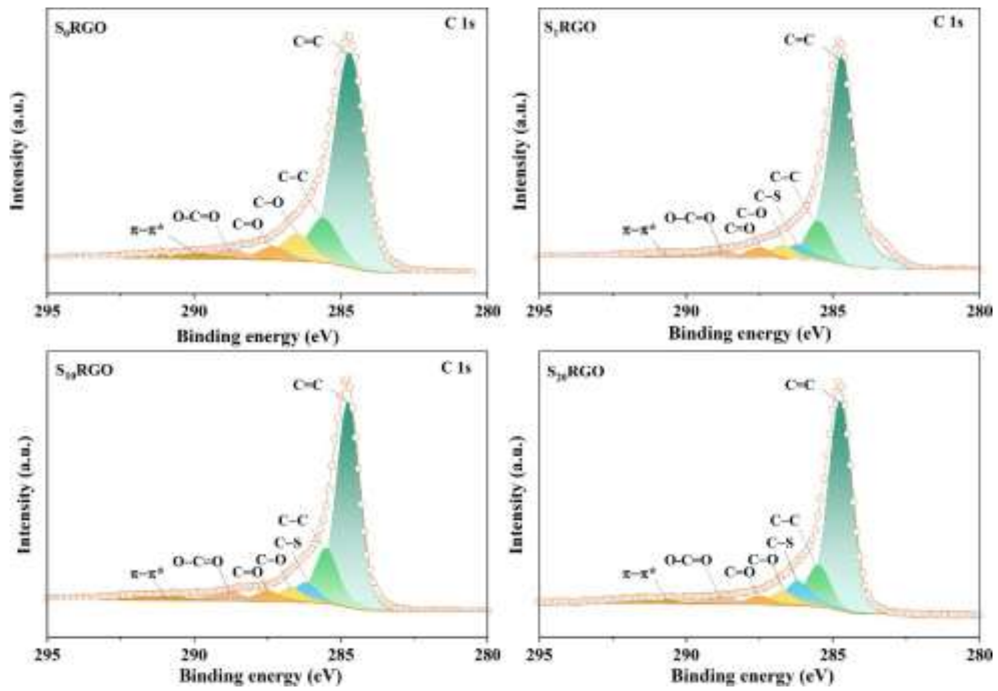

Fig. S10 High-resolution C 1s XPS spectra of S_xRGO.

The C 1s XPS spectrum of S_0RGO can be deconvoluted into distinct six peaks, including the *sp*^{2} C=C (284.7 eV), *sp*^{3} C–C (285.5 eV), C–O (286.7 eV), C=O (287.5 eV), O–C=O (288.8 eV) and π–π* shakeup (291.0 eV) features.[10] For S_xRGO (x = 1, 10, 20), the presence of C–S bonds is also detected at ~286.2 eV in addition to the aforementioned six peaks, indicating that the sulfur exists within the lattice structure of graphene and forms covalent bonds (Table S3).[11]

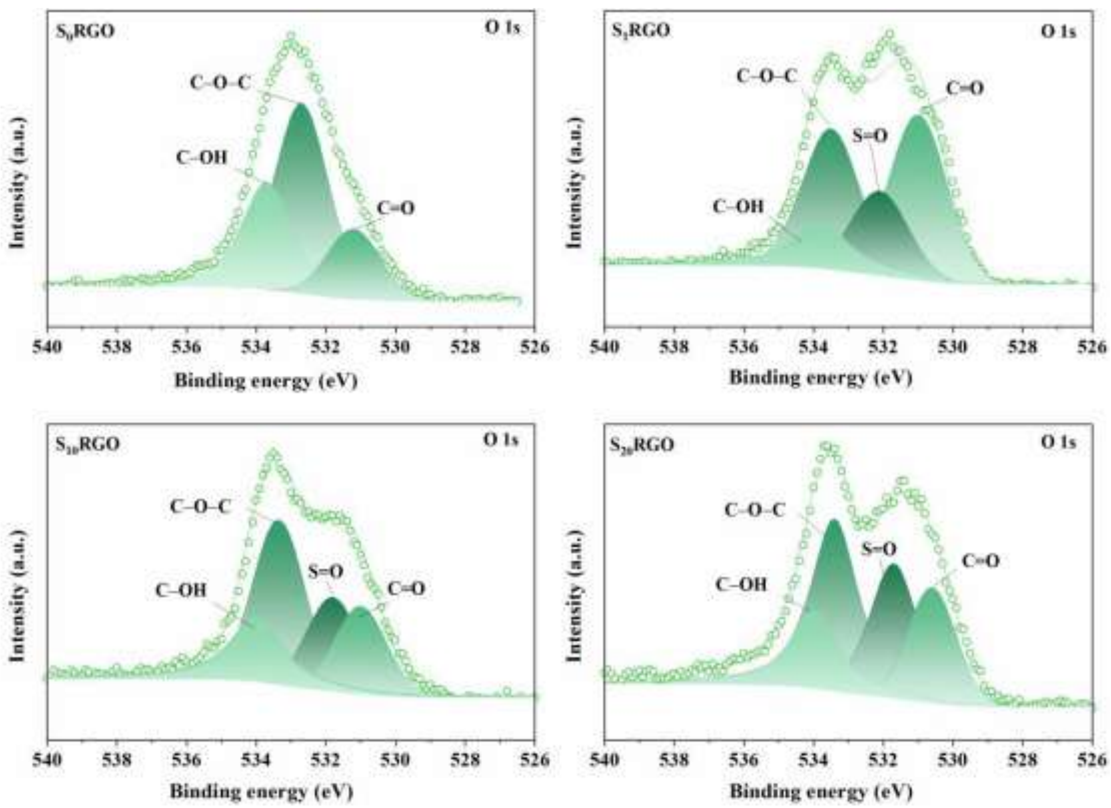

Fig. S11 High-resolution O 1s XPS spectra of S_xRGO.

The oxygen contents of S_xRGO (x = 0, 1, 10, 20) can be obtained to be 17.31, 11.03, 9.21 and 8.79 at%, respectively (Table S2), inferring that deeper reduction of GO occurs with more sulfur doping. In addition, obvious S=O group could be deconvoluted in the O1s XPS spectra of S_xRGO (Table S3).[12]

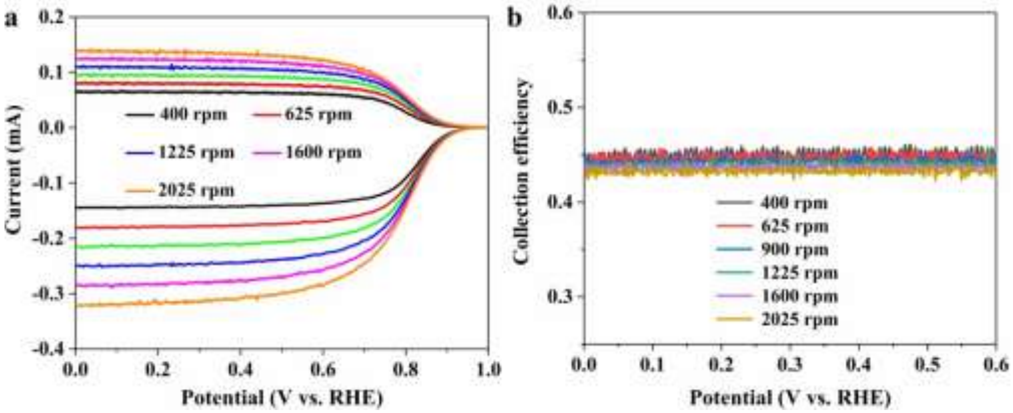

Fig. S12 Calibration of the collection efficiency of the bare RRDE in N_2-saturated 0.05 M Na_2SO_4 dissolved with 4 mM $K_3[Fe(CN)_6]$. Catalyst loading: 143 μg cm^{-2}.

The collection efficiency of the RRDE system is ~0.43.

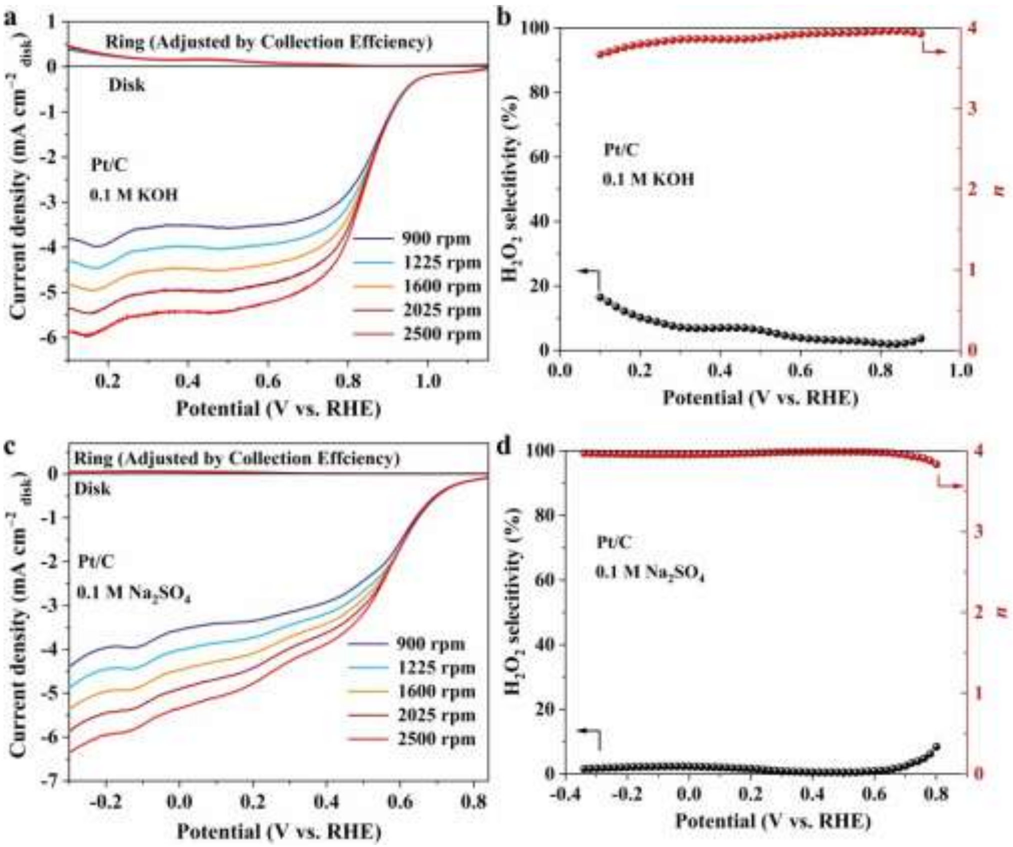

Fig. S13 (a, c) ORR polarization curves of RRDE measurements and (b, d) the corresponding H_2O_2 selectivity and *n* value of Pt/C in O_2-saturated 0.1 M KOH and 0.1 M Na_2SO_4, respectively. Catalyst loading: 143 μg cm^{-2}.

The commercial 20% Pt/C, a well-established 4e^{-} ORR catalyst, was employed as a reference to validate the reproducibility of RRDE.

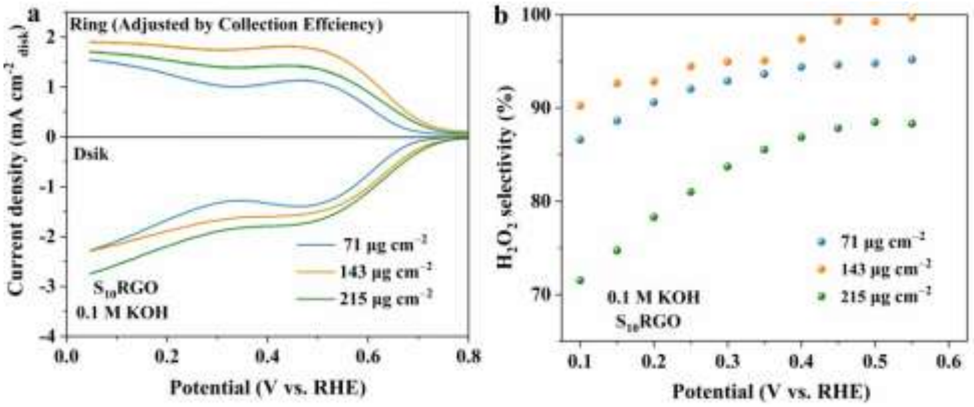

Fig. S14 (a) LSV curves and (b) H_2O_2 selectivity of S_{10}RGO with different loading in O_2-saturated 0.1 M KOH.

The increase in S_{10}RGO loading amount from 71 to 215 μg cm^{-2} leads to a higher disk current density due to more catalytic active sites, resulting in a slightly positive shift in the onset potential. However, the ring current density decreases with further increasing S_{10}RGO loading amount from 143 to 215 μg cm^{-2}, leading to reduced H_2O_2 selectivity. This phenomenon is presumed to be due to the fact that the active sites may be fully saturated by the O_2 flux at the RRDE surface with the low catalyst loading, but overmuch catalytic sites would lead to side reactions (4e$^-$ ORR, H_2O_2 reduction and/or decomposition), reducing H_2O_2 selectivity. Therefore, the optimized catalyst loading of S_{10}RGO was fixed at 143 μg cm^{-2} for all related electrochemical tests.[3,13]

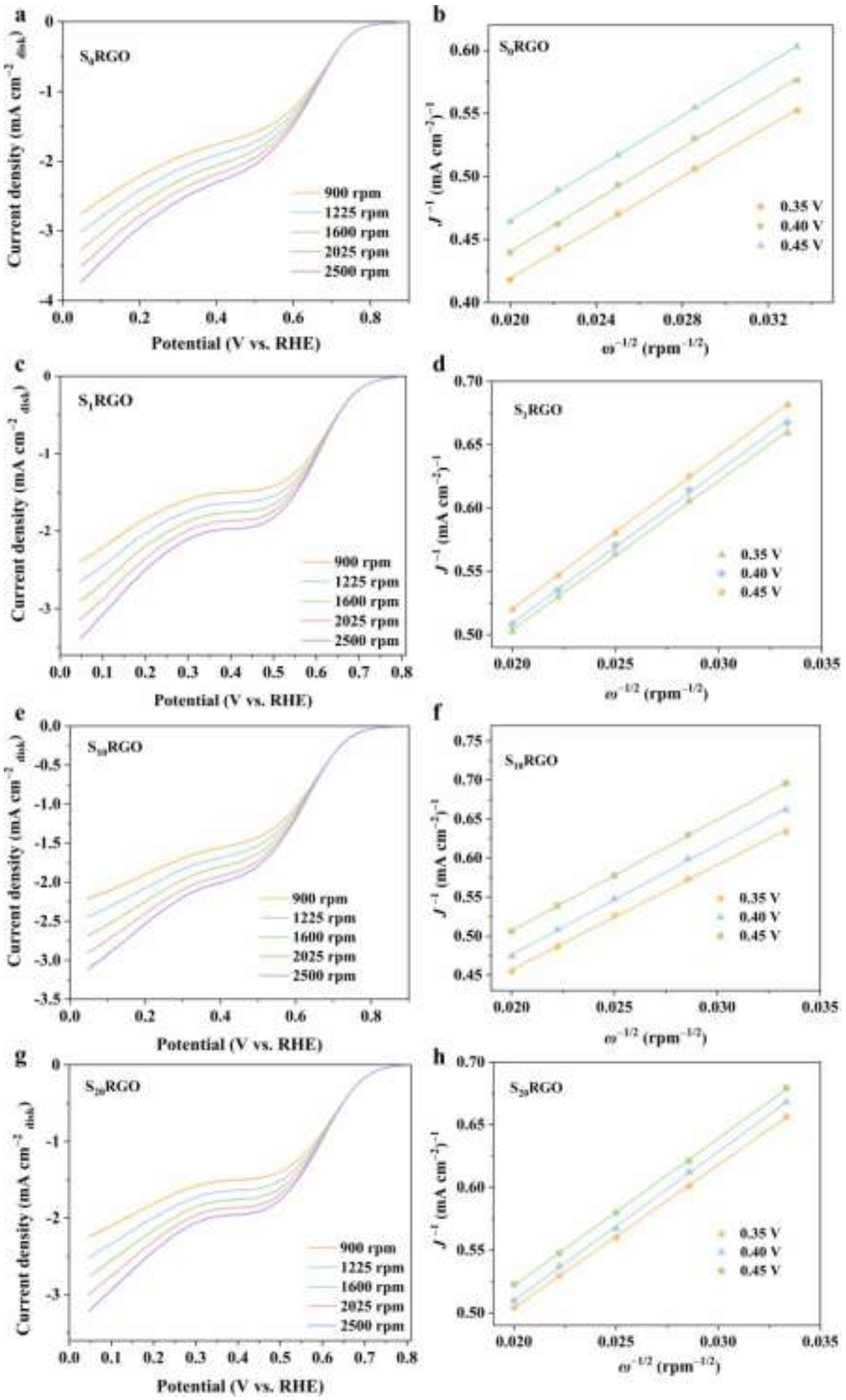

Fig. S15 (a), (c), (e) and (d) electrochemical oxygen reduction polarization curves of S_xRGO (x=0, 1, 10, 20) at different rotation rates in 0.1 M KOH, respectively. (b), (d), (f) and (h) the corresponding K-L plots of S_xRGO (x=0, 1, 10, 20), respectively. Catalyst loading: 143 μg cm^{-2}.

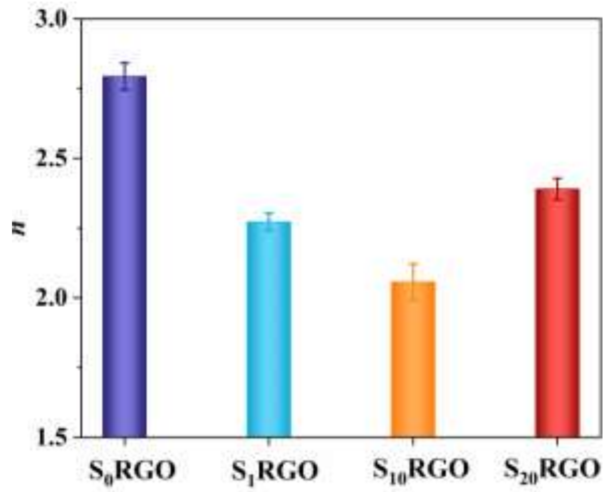

Fig. S16 The electron transfers number (*n*) is based on the K-L plots.

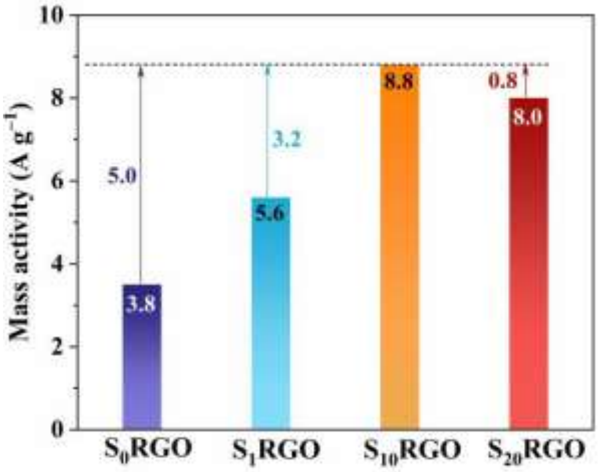

Fig. S17 Mass activity of S_xRGO (x=0, 1, 10, 20) at 0.75 V.

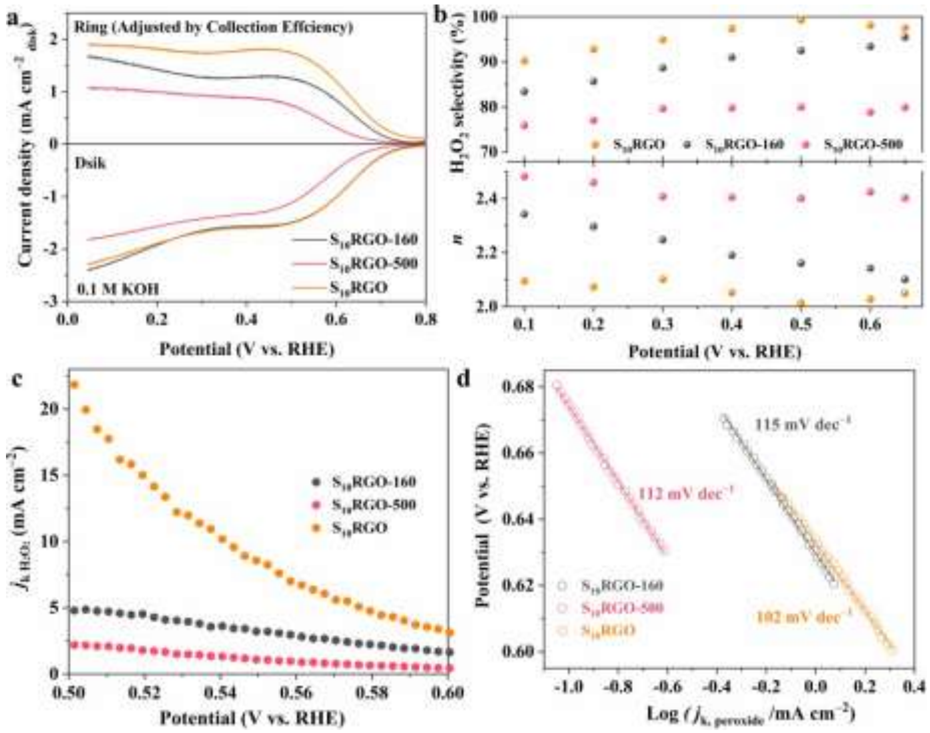

Fig. S18 (a) LSV curves, (b) H_2O_2 selectivity and electron transfer number and (c) j_k at different potentials and (d) Tafel plots of S_{10}RGO-160, S_{10}RGO-500 and S_{10}RGO in 0.1 M KOH. Catalyst loading: 143 μg cm^{-2}.

As shown in Fig. S18a–b, among all the materials, S_{10}RGO exhibited the best catalytic activity toward $2e^-$ ORR, with an early onset potential of 0.773 V. Upon temperature adjustment, the onset potential dropped to 0.724 V and 0.581 V for S_{10}RGO-160 and S_{10}RGO-500, respectively. The S_{10}RGO exhibits an H_2O_2 selectivity of 90%–99.8% in a wide potential range from 0.10 to 0.65 V. Comparatively, the S_{10}RGO-160 and S_{10}RGO-500 produce the H_2O_2 selectivity of 83%–95% and 76%–80% from 0.10 to 0.65 V, respectively. Also, the S_{10}RGO has the highest kinetic current of H_2O_2 and the lowest Tafel slope (Fig. S18c–d). Notably, the kinetic current of H_2O_2 for S_{10}RGO is about 4.4 times higher than that of S_{10}RGO-160 at 0.5 V (Fig. S18c), which indicates that the introduction of more C–S groups in S_{10}RGO obviously enhances the ORR kinetics (Fig. S2). S_{10}RGO-500 underperformed in terms of both activity and selectivity during the $2e^-$ ORR, which may be attributed to the detrimental effects of thiol (–SH) groups, as illustrated in Fig. S3. Consequently, the configuration and composition of sulfur play crucial roles in the $2e^-$ ORR performance of carbon-based materials.

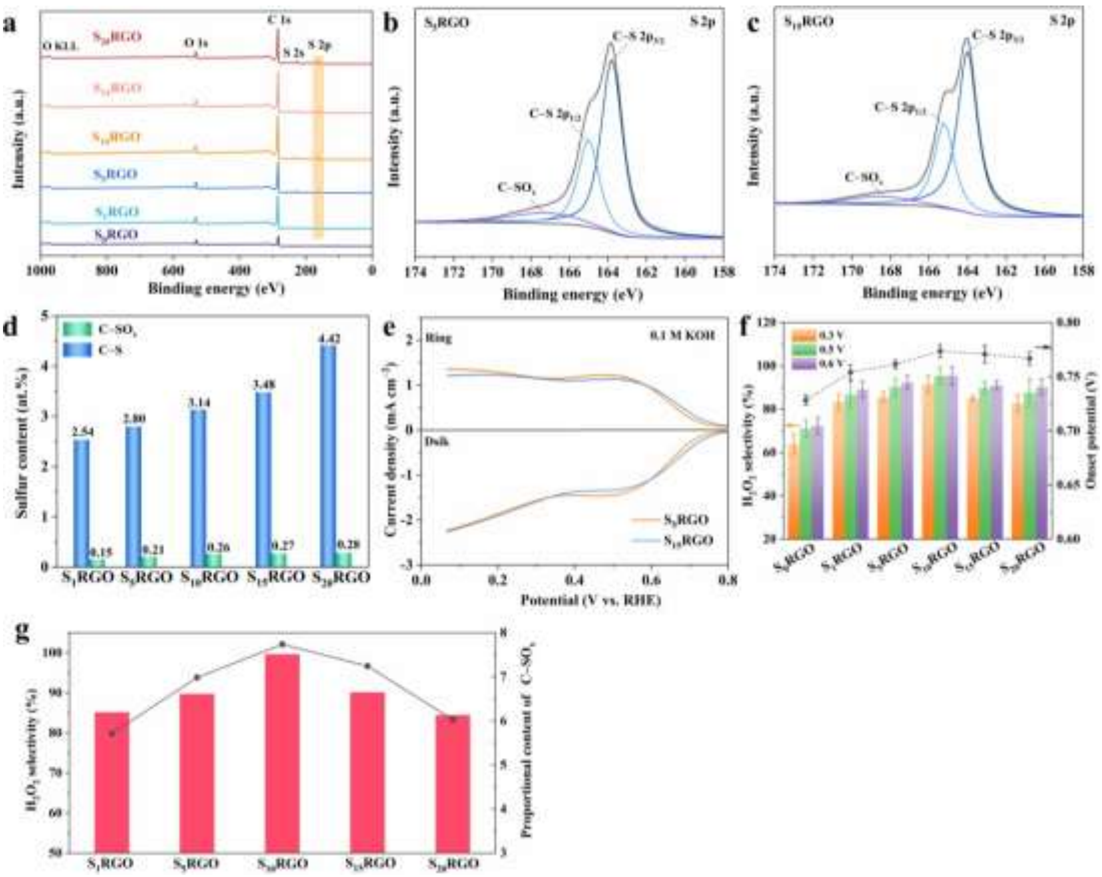

Fig. S19 (a) Survey XPS spectra of S_xRGO. (b) S 2p XPS spectra of S_5RGO. (c) S 2p XPS spectra of $S_{15}RGO$. (d) Proportional content of each sulfur configuration. (e) LSV curves of S_5RGO and $S_{15}RGO$ in O_2-saturated 0.1 M KOH. (f) The H_2O_2 selectivity at different potentials and the onset potential of S_xRGO (x = 0, 1, 5, 10, 15, 20). (g) Dependence of H_2O_2 selectivity and proportional content of C–SO_x. Catalyst loading: 143 μg cm^{-2}.

In order to further verify the catalytic trend, two additional samples designated as S_5RGO and $S_{15}RGO$ were prepared with the similar method with that of S_xRGO (x = 1, 10, 20) except using GO and sublimed sulfur with mass ratios of 1:5 and 1:15 as precursors, respectively. The surface elements and their chemical states of S_5RGO and $S_{15}RGO$ were characterized by XPS analysis (Fig. S19a–c). As presented in Table S3, the atomic sulfur content in the S_xRGO (x = 1, 5, 10, 15, 20) samples increases proportionally with the rise in the S/GO mass ratio. When the S/GO mass ratio exceeds 10, the enhanced sulfur incorporation is predominantly driven by the formation of C–S bonds, while the generation of –SO_x moieties shows limited growth (Fig. S19d). This

behavior can be attributed to the fact that –SO_x functional groups primarily form at the edge defects of GO, which will approach saturation at higher sulfur loadings, thereby significantly limiting the further formation of –SO_x species.

The electrocatalytic ORR performances of S_5RGO and S_{15}RGO were also assessed (Fig. S19a) and compared with S_xRGO (x = 1, 10, 20). As shown in Fig. S19b, the onset potential and H_2O_2 selectivity orders are S_{10}RGO > S_{15}RGO >S_{20}RGO > S_5RGO > S_1RGO >S_0RGO, which further confirms the previous catalytic trend. In comparison with S_0RGO, all S_xRGO (x = 1, 5, 10, 15, 20) catalysts exhibit higher onset potentials, all of which surpass 0.760 V, indicating that sulfur doping plays a pivotal role in enhancing the 2e⁻ ORR activity. Moreover, as indicated in Fig. S19c, the H_2O_2 selectivity displays a positive correlation with the proportion of C–SO_x content in catalysts.

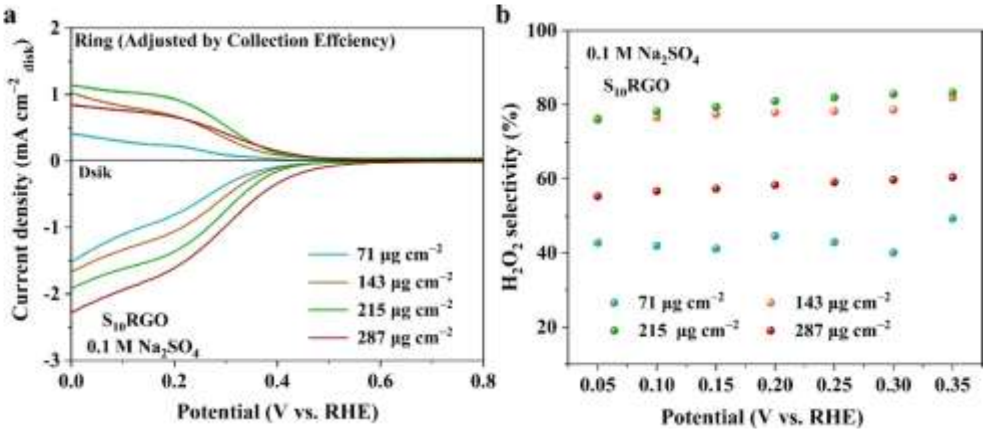

Fig. S20 (a) LSV curves and (b) H_2O_2 selectivity of $S_{10}RGO$ with different loading in O_2-saturated 0.1 M Na_2SO_4.

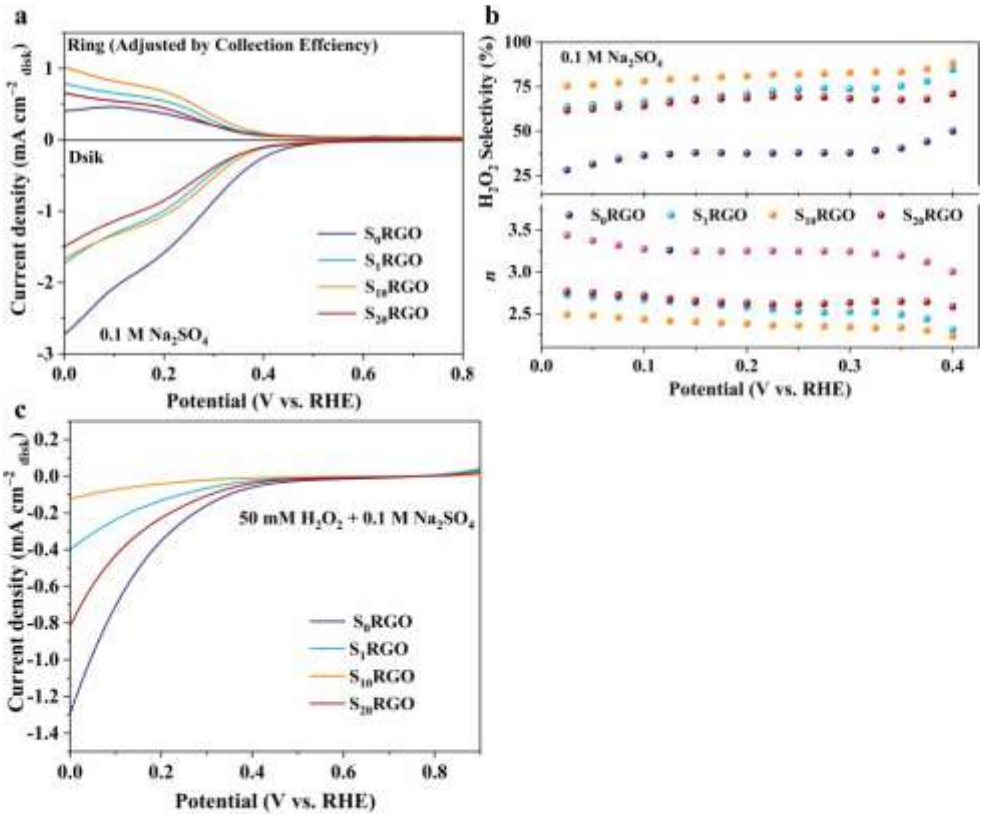

Fig. S21 (a) LSV curves and (b) H_2O_2 selectivity of S_xRGO (x=0, 1, 10, 20) in 0.1 M Na_2SO_4. (c) LSV curves of S_xRGO (x=0, 1, 10, 20) in 0.1 M Na_2SO_4 containing 50 mM H_2O_2. Catalyst loading: 600 μg cm^{-2}.

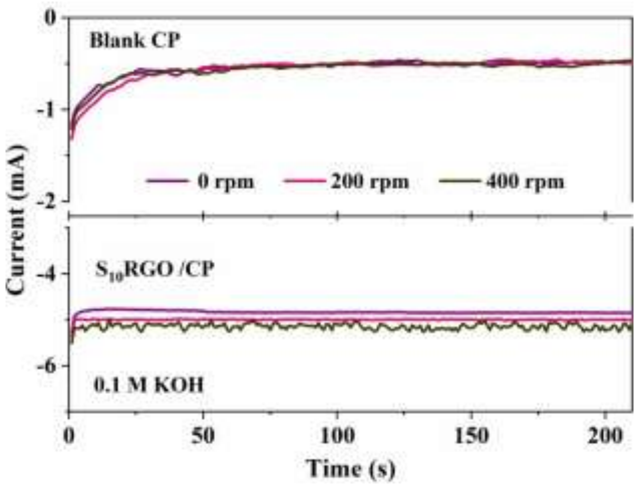

Fig. S22 Chronoamperometry tests of S_{10}RGO/CP and blank CP in O_2-saturated 0.1 M KOH at different stirring rates in the three-electrode H-type cell. Catalyst loading: 600 μg cm^{-2}.

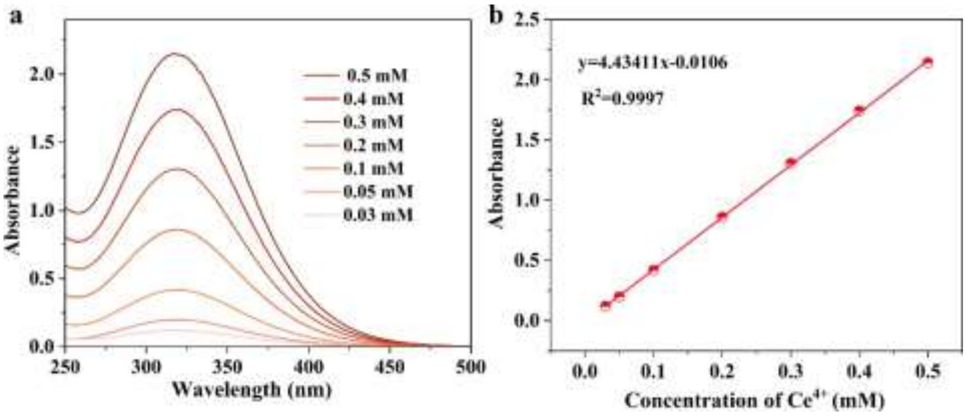

Fig. S23 (a) UV-vis absorption spectra of Ce^{4+} solution with various concentrations and (b) the corresponding standard curve.

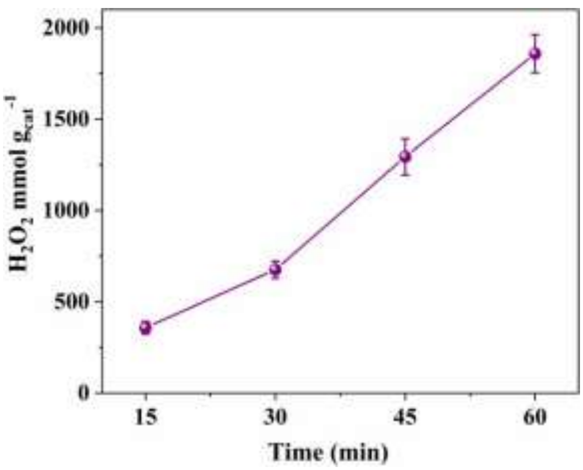

Fig. S24 Accumulation of H_2O_2 over time in O_2-saturated 0.1 M KOH at stirring rate of 400 rpm in the three-electrode H-type cell. Catalyst loading: 600 μg cm^{-2}.

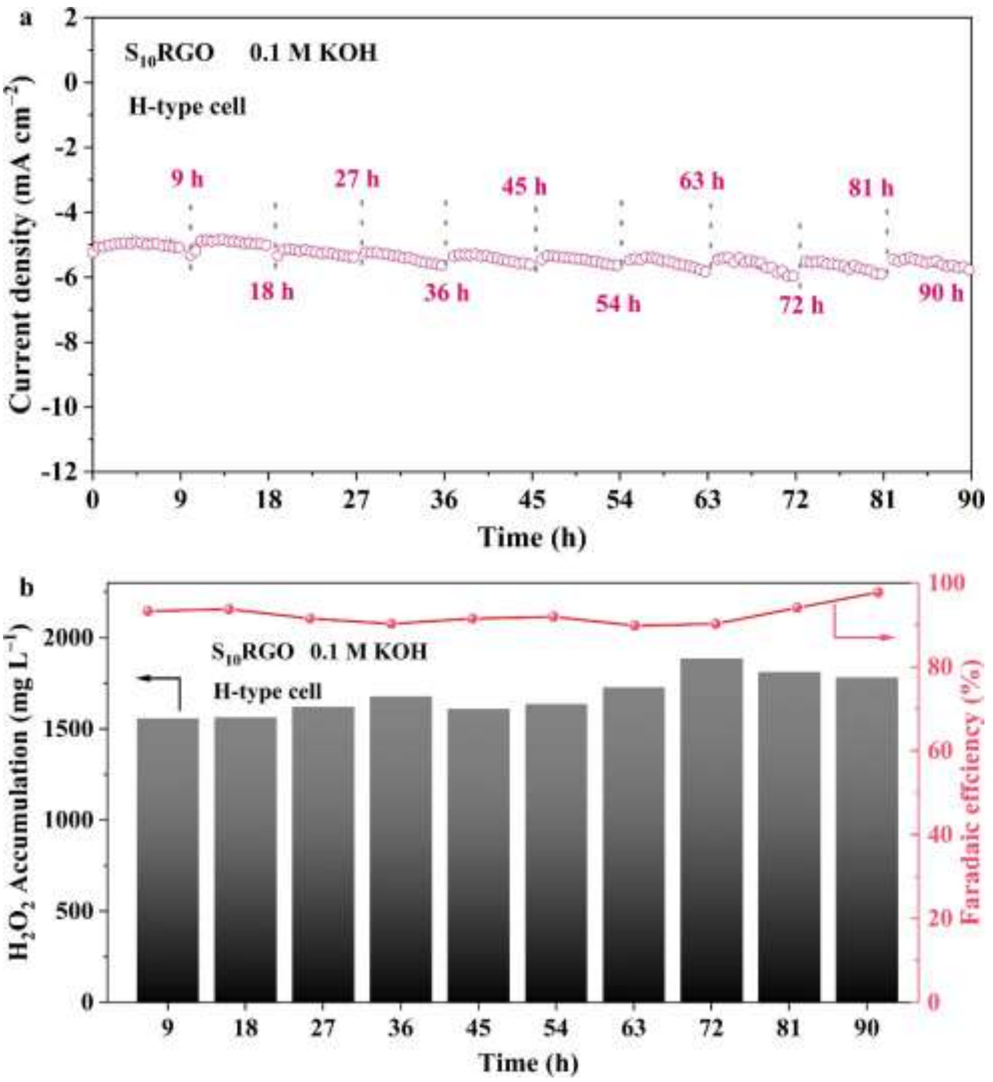

Fig. S25 (a) Long-term stability test of S_{10}RGO at 0.52 V in O_2-saturated 0.1 M KOH over 90 h. (b) Faraday efficiency and H_2O_2 accumulation obtained using S_{10}RGO every 9 h determined via cerium sulfate titration during 90 h at 0.52 V. Catalyst loading: 600 µg cm^{-2}.

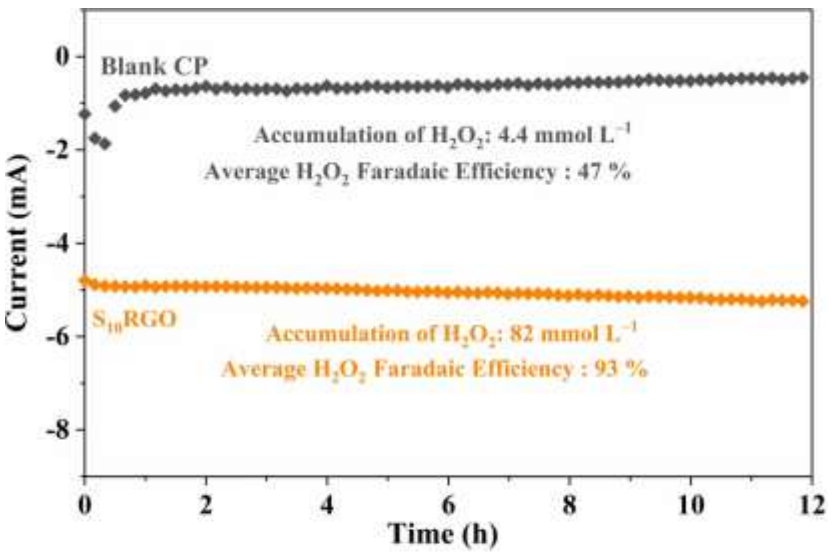

Fig. S26 Chronoamperometry of the blank carbon paper and S_{10}RGO electrode in the H-type cell. Catalyst loading: 600 μg cm^{-2}.

The current density in the H-type cell was increased from 5 mA cm^{-2} to 5.19 mA cm^{-2} after 12 h of testing, as illustrated in Fig. S26. The slight increase in the current during the stability test is attributed to two possible factors: first, the electrochemically active area may have expanded due to the diffusion of the reaction substrate during electrolysis. Second, the gradual accumulation of H_2O_2 formed in the electrolysis process could contribute to this increase. It has been reported that H_2O_2 can elevate the potential of the reference electrode, thereby increasing the actual electrolytic potential and improving the electrolytic current.[14] As depicted in Fig. S25a, when the electrolyte is refreshed every 9 h, the current density will revert to its nearly initial value.

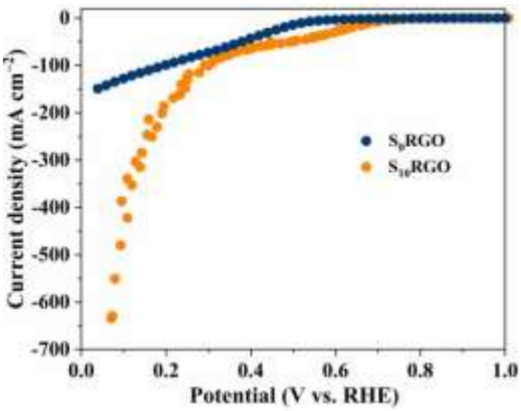

Fig. S27 LSV curves of S_0RGO and $S_{10}RGO$ tested under O_2 atmospheres in the flow-cell setup, respectively. Catalyst loading: 600 µg cm^{-2}.

To understand the unexpected shape of the polarisation curve, the LSV curves of the catalyst in the conventional three-electrode system was carefully observed again. As illustrated in Fig. 2a, unlike the S_0RGO catalyst that shows the decreased ring current density at potentials below 0.3 V, the sulfur-doped catalyst especially for $S_{10}RGO$ shows a slightly increased ring current density below 0.3 V, indicating that the sulfur-doped catalyst can inhibit the decomposition of H_2O_2 in the low potential region. Similarly, the LSV curve in the flow cell actually reflects a similar current variation trend, showing increased current at below 0.3 V.

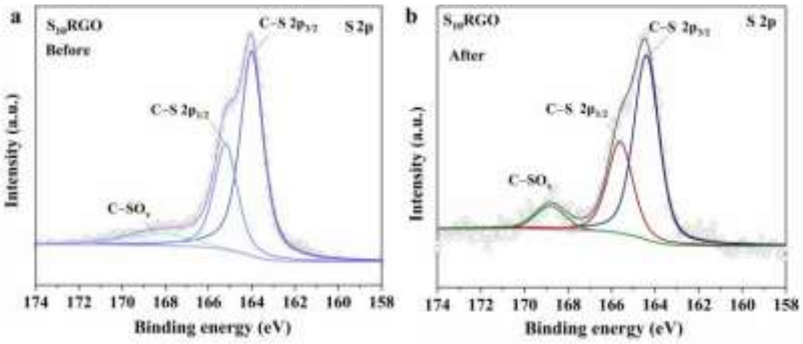

Fig. S28 High-resolution S 2p XPS spectra of $S_{10}RGO$ before and after LSV curve measurements in the flow cell.

The XPS spectra of $S_{10}RGO$ were obtained to study the structural features before and after LSV scanning. The results show that both C–S and C–SO_x bonds are present on the catalyst surface before and after the LSV tests, but some of the C–S bonds have been oxidized to C–SO_x species after the LSV test, which is likely due to the oxidative processes that occur during H_2O_2 generation. As is known, the ORR is carried out with

H_2O as the proton source in alkaline electrolytes, where H_2O is involved in participating in the proto-coupled electron transfer (PCET) process. A recent study has proven that adding dimethyl sulfoxide (DMSO) to KOH electrolytes can enhance H_2O_2 selectivity by constructing H_2O-DMSO hydrogen-bonding networks to alter proton transfer kinetics.[15] This sheds light on the understanding the above phenomenon in our experiment. It is speculated that the incorporation of SO_x group on the catalyst surface would affect the interfacial microenvironment of the catalyst, in which SO_x group would form hydrogen bond with H_2O, thereby affecting the mass transfer process. As a result, the H* supply to participate in the ORR is reduced, hindering the excessive hydrogenation of H_2O_2 to H_2O ($OOH^* + H^+ + e^- \rightarrow O + H_2O$) and promoting the formation of H_2O_2.

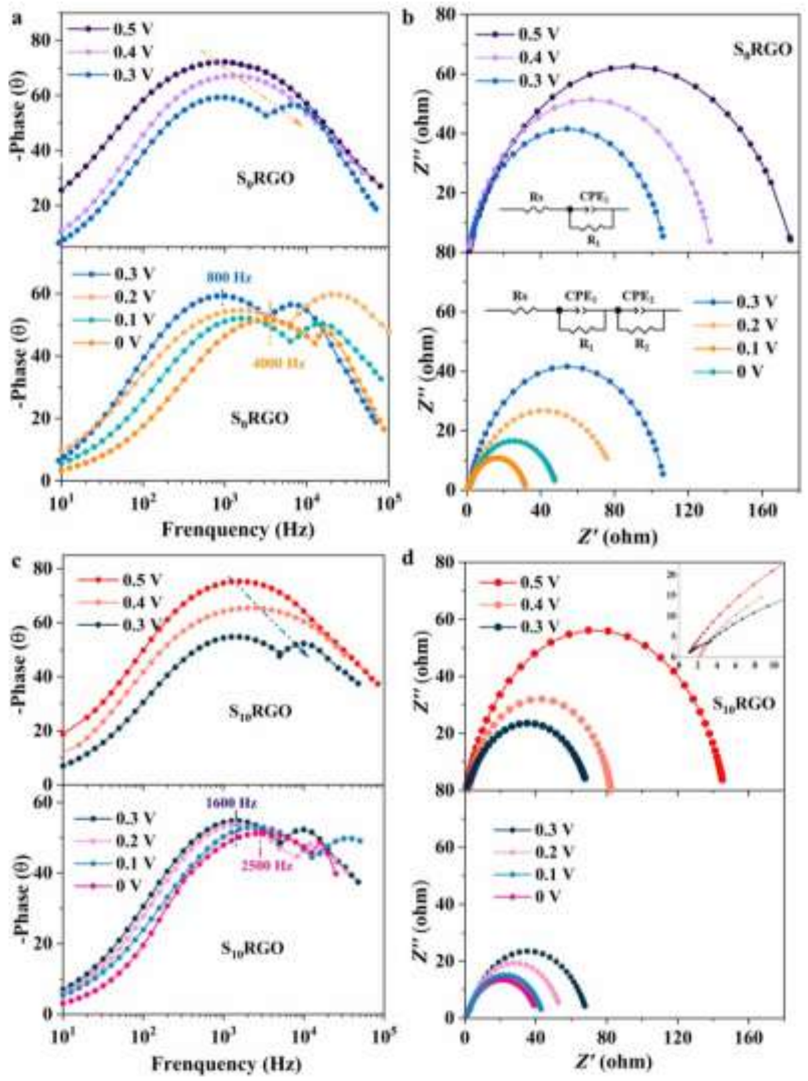

Fig. S29 Bode plots (a, c) and Nyquist plots (b, d) at 0.5–0 V for S_0RGO and S_{10}RGO. The inset in (b) is the equivalent circuit used to fit the Nyquist plots, and (d) shows the magnified images of the low-frequency region.

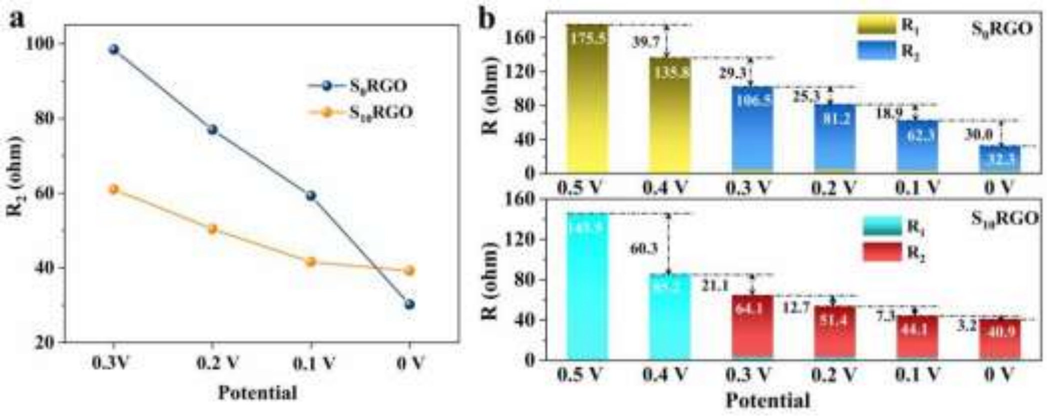

Fig. S30 (a) The mass transfer resistance (R_2) at 0.3–0 V and (b) resistance at 0.5–0 V. The Resistance (R) is the sum of R_1 and R_2.

As shown in Fig. S29, in the range of 0.5–0.3 V, similar changes of EIS were presented in S_0RGO and S_{10}RGO. With increasing bias potential (0.5 V to 0.3 V), the arc diameter of the Nyquist plots decreases and phase angle ($Ø_{peak}$) of the Bode plots shifts to high frequency. Equivalent circuit fitting revealed a rapid decline in charge transfer resistance (R_1) with increasing bias potential, confirming the ORR is mainly controlled by the charge transfer process.

When the bias reaches to 0.3 V, a clear double-peak feature can be seen in the Bode plot, indicating the existence of two charge transfer processes at the interface. The Nyquist plot shows a new arc, related to the mass transfer process. A new equivalent circuit diagram (the inset in Fig. S29b) with a mass transfer capacitance (CPE_2) and mass transfer resistance (R_2) was applied. In this situation, the ORR is jointly controlled by the charge transfer and mass transfer processes.[16,17]

In the potential range of 0.3–0 V, the ORR pathway demonstrates predominant mass transport control. For the S_0RGO catalyst, Bode phase angle analysis reveals a significant $Ø_{peak}$ decrease accompanied by peak frequency shifts toward higher frequency domains (Fig. S29a). This trend coincides with a marked reduction of semicircle diameters in Nyquist plot (Figs. S29b and S30). These results indicate enhanced mass transfer kinetics. By contrast, the S_{10}RGO catalyst displays distinct electrochemical behavior. Slowed $Ø_{peak}$ attenuation and diminished high-frequency shifts are observed in Bode plots in comparison to S_0RGO (Figs. S29c and S29d).

Nyquist analysis further reveals gradual mass transport resistance reduction with increasing applied potential (Fig. S30). This gradual resistance reduction suggests moderated mass transport kinetics, potentially originating from sulfoxide group-water molecular interactions at the electrode-electrolyte interface. Such retarded mass transfer characteristics create favorable interfacial conditions for selective H_2O_2 synthesis through inhibiting the attack of active hydrogen to H_2O_2.[16,17]

As a consequence, the unexpected shape of the polarisation curve in the flow cell may be attributed to the following potential factors. During the accumulation of H_2O_2 during 0.5–0.3 V, some of the C–S bonds were oxidized to C–SO_x species, which would influence the interfacial interaction between catalyst and H_2O, thereby affecting the mass transfer process, inhibiting the attack of active hydrogen to H_2O_2, thus inhibiting the decomposition of H_2O_2 and promoting the formation of H_2O_2.

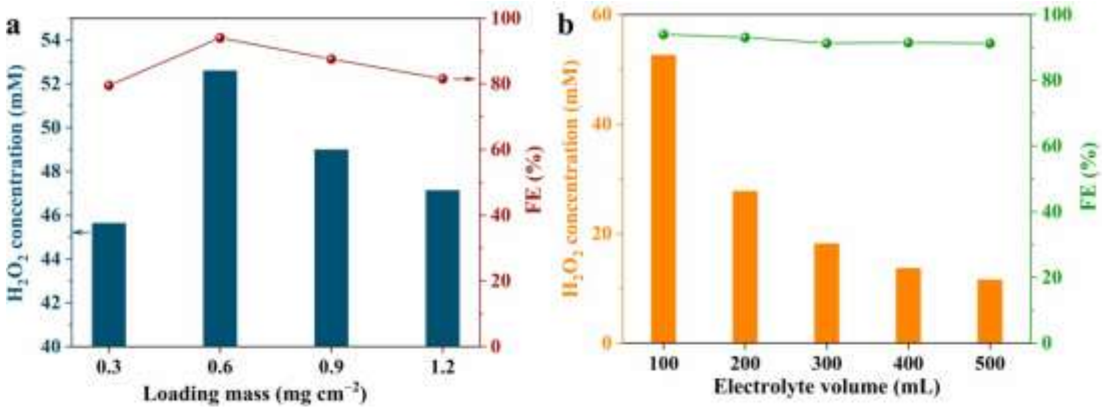

Fig. S31 The accumulated H_2O_2 concentrations and FE of S_{10}RGO in the flow cell at 300 mA cm^{-2} for 1 h in relation to (a) load mass and (b) volume of electrolyte.

Optimization experiments concerning catalyst loading and electrolyte volume were performed in an alkaline flow cell at 0.15 V, achieving a current density of 300 mA cm^{-2} for a duration of 1 h. To stabilize the produced H_2O_2, 10 mM EDTA was incorporated into the electrolyte solution. The data presented in Fig. S31a illustrate an initial rise followed by a decline in both H_2O_2 concentration and Faraday efficiency with increasing catalyst load mass. This trend could stem from the saturation of active sites on the carbon paper surface at low catalyst loadings, whereas excessive catalytic sites may lead to side reactions, such as 4e$^-$ ORR, H_2O_2 reduction, and/or decomposition, thereby diminishing H_2O_2 selectivity. Consequently, the optimal catalyst loading for S_{10}RGO was determined to be 0.6 mg cm^{-2}.

Furthermore, as depicted in Fig. S31b, the H_2O_2 concentration exhibited a gradual decline with an increase in electrolyte volume. A smaller electrolyte volume is beneficial in achieving higher concentrations of H_2O_2.[3] It is noteworthy that the S_{10}RGO catalyst maintained a stable H_2O_2 Faradaic efficiency exceeding 90% during the ORR at a current density of 300 mA cm^{-2} across various electrolyte volumes, further underscoring its exceptional H_2O_2 selectivity in practical ORR applications. In summary, the optimal electrolyte volume was identified as 100 mL, yielding a H_2O_2 accumulation concentration of 52 mM for 1 h and achieving a Faraday efficiency of 93.98%.

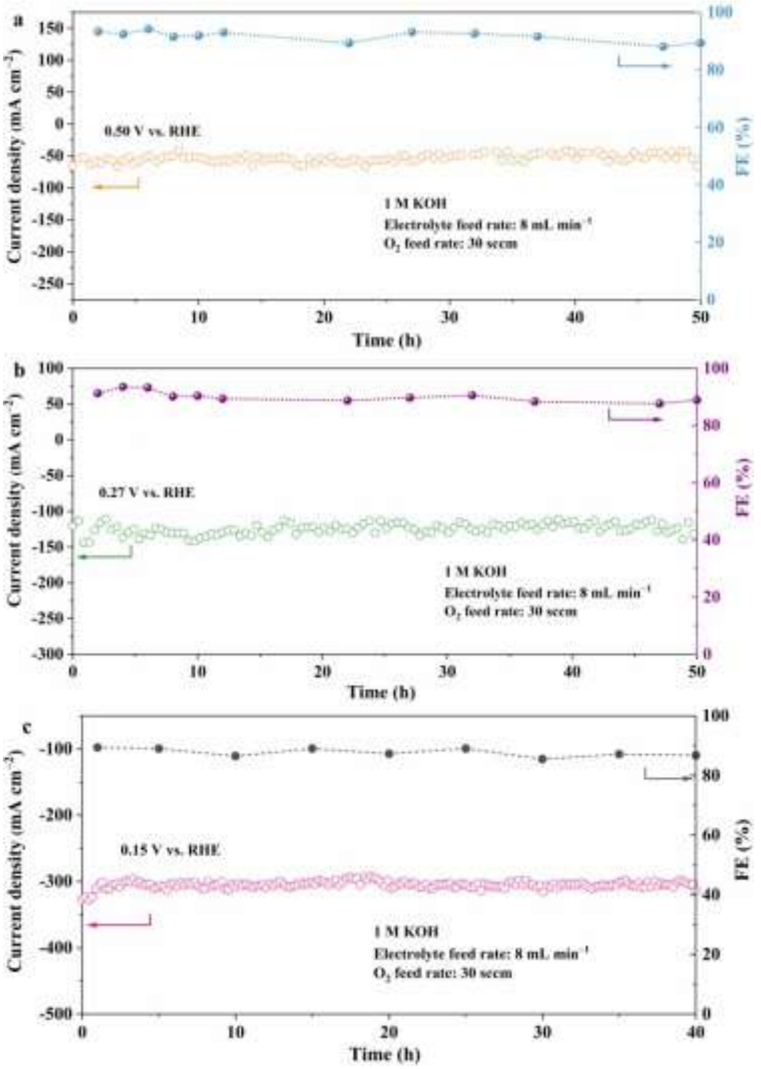

Fig. S32 Chronoamperometry test of S_{10}RGO at different potentials for H_2O_2 production in the flow cell using 1.0 M KOH as electrolyte and the FE. The electrolyte refreshed every 10 h during the test. Catalyst loading: 600 μg cm^{-2}.

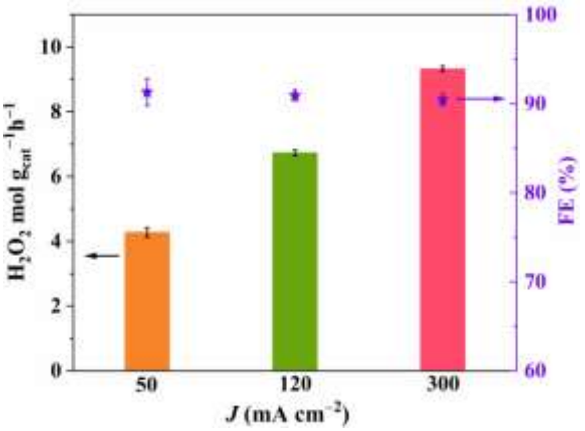

Fig. S33 The H_2O_2 production rate and FE of $S_{10}RGO$ in the flow cell at different potentials. Catalyst loading: 600 μg cm^{-2}.

Significantly, during the bulk electrolysis at 0.50 V and 0.27 V, a high current densities of 50 mA cm^{-2} and 120 mA cm^{-2} can be consistently maintained without noticeable degradation for at least 50 h, respectively. Moreover, at an industrial current density of 50 and 120 mA cm^{-2}, the $S_{10}RGO$ exhibited an average H_2O_2 Faradaic efficiency of approximately 91.3% and 91.0% and achieved a high production rate of 4.28 ± 0.15 mol $g_{catalyst}^{-1}$ h^{-1} and 6.74 ± 0.18 mol $g_{catalyst}^{-1}$ h^{-1}, respectively. These results suggest that $S_{10}RGO$ is a promising candidate for H_2O_2 electrosynthesis at the industrial level.

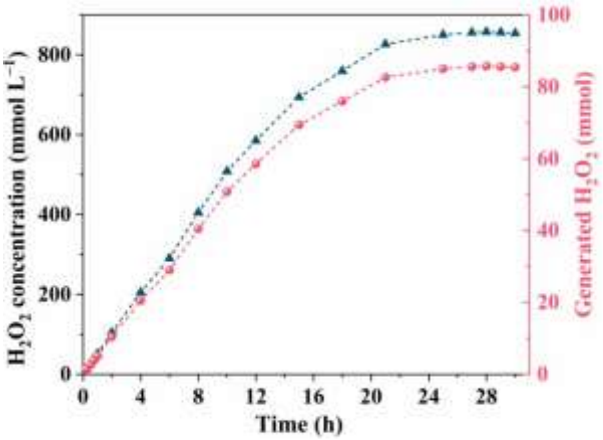

Fig. S34 Accumulation of H_2O_2 over time in flow cell at a potential of 0.15 V. Catalyst loading: 600 μg cm^{-2}.

The long-term accumulation of H_2O_2 at a potential of 0.15 V over a period of 29 h was executed in a flow cell containing 100 mL of alkaline electrolyte, as illustrated in Fig. S34. Initially, the concentration of accumulated H_2O_2 displayed an almost linear escalation during the first 10 h. Followingly, from 10 to 25 h, the rate of H_2O_2 accumulation began to slow down, gradually approaching a state of equilibrium. Throughout this interval, the cumulative amount of H_2O_2 reached a significant 857.5 mmol L^{-1} (equivalent to 3.0 wt%), which is deemed adequate for the production of medical-grade disinfectants. Beyond the 28 h mark, the formation of H_2O_2 decelerated, attributable to its further reduction to H_2O or chemical decomposition into O_2.

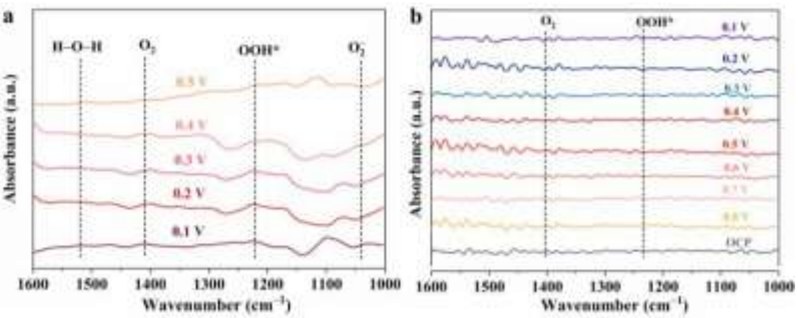

Fig. S35 ATR-SEIRAS spectra recorded on $S_{10}RGO$ at potential range in O_2-saturated (a) 0.10 M Na_2SO_4 and (b) 0.1 M KOH.

Under neutral conditions, two characteristic absorption peaks at ~1240 cm^{-1} (O–O stretching of OOH*) and ~1420 cm^{-1} (O–O stretching of adsorbed O_2) emerged at potentials ≤0.4 V, with intensities increasing at lower potentials, confirming the accumulation of critical intermediates. The intact O–O bond in OOH* suggested a dominant $2e^-$ pathway for H_2O_2 production. Similarly, under alkaline conditions, analogous peaks at 1234 cm^{-1} (OOH*) and 1401 cm^{-1} ($O_{2,ad}$) were observed, reaffirming the persistence of the OOH* intermediate across pH environments. These results collectively demonstrate that $S_{10}RGO$ sustains a selective $2e^-$ ORR pathway for H_2O_2 generation over a broad pH range, with direct spectral evidence highlighting the critical role of the OOH* intermediate in both systems.

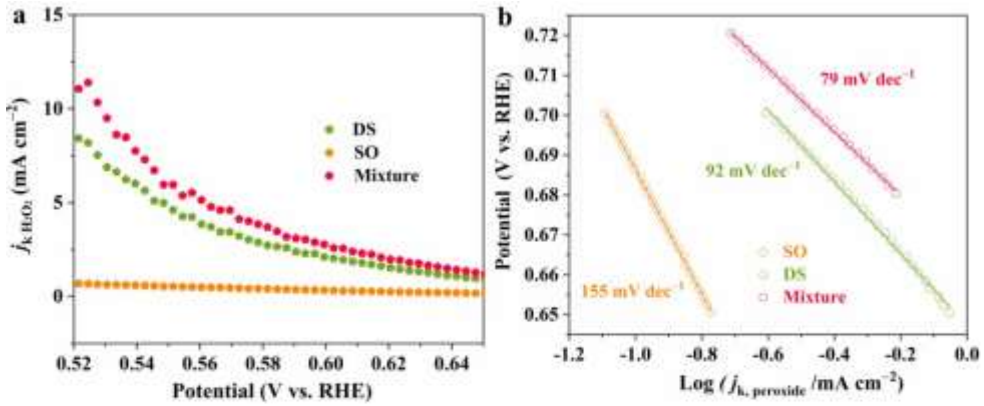

Fig. S36 (a) j_k at different potentials and (d) Tafel plots of dibenzothiophene (DS), sulfobenzide (SO) and their mixture in 0.1 M KOH.

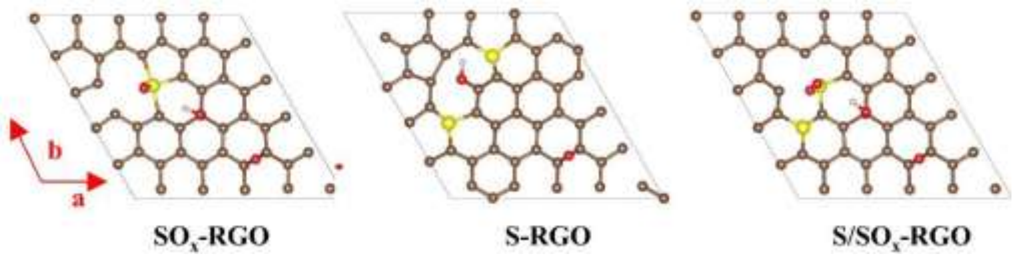

Fig. S37 Atomic structures of SO$_x$-RGO, S-RGO and S/SO$_x$-RGO. The white, brown, red, and yellow spheres denote for H, C, O, and S atoms.

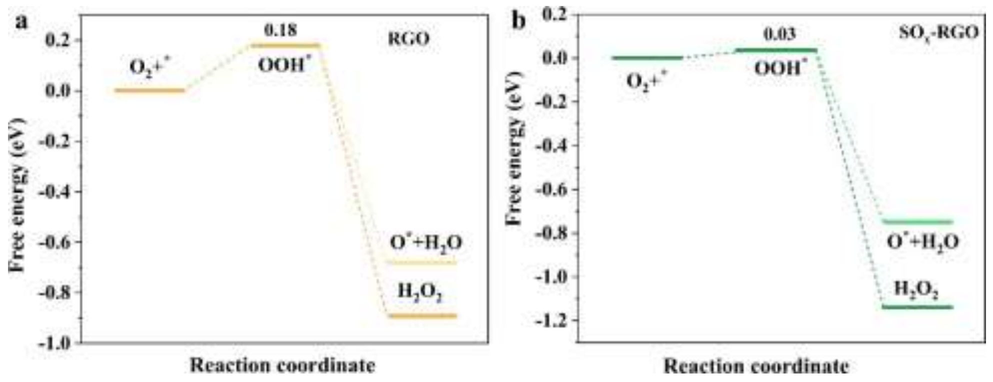

Fig. S38 The free energy diagrams for (a) RGO and (b) SO$_x$-RGO during 2e$^-$ and 4e$^-$ ORR.

Table S1. Raman analysis of S_xRGO (x = 0, 1, 10, 20).

Sample	FWHM		Position		I_D/I_G ratio
	D	G	D	G	
S_0RGO	189.94	100.92	1361.01	1586.81	0.92
S_1RGO	140.15	94.65	1357.40	1586.22	1.12
$S_{10}RGO$	145.59	99.89	1357.36	1586.66	1.13
$S_{20}RGO$	146.40	96.96	1360.10	1588.53	1.15

Raman spectroscopy is a powerful tool to reflect the defect and disorder level of carbonaceous materials.[18] The degree of disorder in the structure can be evaluated by the intensity ratio of D band and G band (I_D/I_G), where D band is related to the defects in sp^2 lattice and G band is related to graphitic structure. A high I_D/I_G ratio means a high number of defect sites. Meanwhile, there was a reduction in the full width at half maximum (FWHM) of both D and G bands in S_xRGO (x = 1, 10, 20) when compared with that of S_0RGO.

Table S2. C_{dl} and corresponding ECSA of S_xRGO (x= 0, 1, 10, 20).

Catalyst	C_{dl} ($\mu F\ cm^{-2}$)	ECSA ($cm^2\ cm_{electrode}^{-2}$)
S_0RGO	741	18.53
S_1RGO	837	20.93
$S_{10}RGO$	874	21.85
$S_{20}RGO$	843	21.08

Table S3. Atomic content of C, O and S for S_xRGO (x = 0, 1, 5,10, 15, 20) according to XPS survey spectra.

Sample	C (at%)	O (at%)	S (at%)	C/O ratio
S_0RGO	82.9	17.31	-	4.78
S_1RGO	86.28	11.03	2.69	7.82
S_5RGO	86.68	10.31	3.01	8.41
$S_{10}RGO$	87.41	9.21	3.37	9.49
$S_{15}RGO$	87.29	8.96	3.75	9.72
$S_{20}RGO$	86.36	8.79	4.65	9.82

Moreover, the atomic ratio of sulfur in S_xRGO (x = 0, 1, 5,10, 15, 20) increased with the increasing ratio of sublimed sulfur and GO during synthesis of S_xRGO.

.

Table S4. EA data of the S_xRGO (x = 0, 1, 10, 20).

Sample	EA (wt%)
S_0RGO	-
S_1RGO	7.8 ± 0.08
$S_{10}RGO$	8.2 ± 0.03
$S_{20}RGO$	9.9 ± 0.08

Table S5. Atomic content of different bonds for S_xRGO (x = 0, 1, 10, 20) according to S 2p, C 1s and O 1s XPS spectrum.

Sample	S 2p (at%)		C 1s (at%)	O 1s (at%)
	C–S	C–SO_x	C–S	S=O
S_1RGO	2.53	0.15	5.05	1.86
$S_{10}RGO$	3.13	0.26	6.00	1.98
$S_{20}RGO$	4.41	0.28	9.00	2.16

Table S6. Electrocatalytic performances for H_2O_2 production by $S_{10}RGO$ and various reported catalysts via 2e⁻ ORR in alkaline media.

Classification	Catalyst	Electrolyte (0.1 M)	Potential (V vs. RHE)	Selectivity (%)	Catalyst Loading (mg cm⁻²)	Production H-type cell, (mmol gcat⁻¹ h⁻¹)
Carbon materials	$S_{10}RGO$ This work	KOH	0.1–0.65	90─98.9	0.143	1853.85 ± 300
	rGO/PEI[19]	KOH	0.74	90.7	0.100	106.4
	O-GOMC[20]	KOH	0.2–0.7	< 90	0.050	/
	O-xoG[21]	KOH	0.2	82	0.100	224.8
	N-mFLG-8[22]	KOH	0.3–0.7	95–98.5	0.101	/
	$OCNS_{800}$[23]	KOH	0.55–0.75	90–91	0.100	/
	CNB-ZIL-8 [24]	KOH	0.2–0.6	80–85	0.125	/
	NCMK3IL50_800T[25]	KOH	0.4	86	0.050	561.7
	HPCS-S[26]	KOH	0.3	70	/	183.99
	NSMC-0.2[27]	KOH	0.2	92	0.204	/
	thiophene-S[28]	KOH	0.5–0.75	< 90	0.303	/
	S-Nv-C_3N_4[29]	KOH	~0.35	95	0.161	4520
	B-C[30]	KOH	0.65	90	0.1	/
	S-DNC[31]	KOH	0.78	90	/	690 mg L^{-1} h^{-1}
	S-mC-0.375[32]	KOH	0.2–0.7	92–99%	0.2	/
Single-atom metal catalysts	Co-N-C[33]	KOH	0.3	68	0.100	/
	Co_1-NG(O)[34]	KOH	0.1	82	0.010	418±19
	Ni-SA/G-O[35]	KOH	0.1–0.5	< 94	0.051	/
Nobel metal catalysts	Pt-Hg[36]	KOH	0.2–0.4	< 96	0.029	/
	Pb-SA/OSC[37]	KOH	0.3–0.7	90–94	0.121	/

Table S7. Performance parameters of $S_{10}RGO$ and various reported catalysts.

Catalyst	Mass activity at 0.75 V ($A\ g^{-1}$)	Potential range of H_2O_2 Selectivity >90%	Stability
$S_{10}RGO$<br>This work	8.8[a]<br>22.5[b]	0.1–0.65 V	[d]40 h@300 mA cm^{-2}
Co-N_5-O-C[38]	87.5[b]	the maximum H_2O_2 selectivity was 85.6%	[d]24 h@200 mA cm^{-2}
Co-N_2-C/OH[39]	24.25[b]	0.45–0.75 V	/
HCNFs[40]	80.5[b]	0.2–0.7 V	/
Co_1-NG (O)[34]	66[b]	the maximum H_2O_2 selectivity was 82%	[c]110 h@ 5 mA cm^{-2}
$OCNS_{900}$[23]	14.5[a]	0.55–0.75	[d]11 h@50 mA cm^{-2}
GNPC=O,1[14]	5.2[a]	0.5–0.78	[c]120 h@ 1 mA
O-GOMC-x[41]	9.6[a]	0.2–0.7 V	[c]16 h@ 5 mA cm^{-2}
O-CNTs[42]	3.5[a]	0.45–0.65 V	/
MCHS-9:1[43]	~4.7[a]	0.35–0.62 V	/
BN-C1[44]	3.2[a]	the maximum H_2O_2 selectivity was 85%	/

a Mass activity was calculated with the actual current from LSV curves.

b Mass activity was calculated with the kinetics current and have excluded the mass transport effect.

c The stability test was conducted in a flow cell device in an alkaline solution.

d The stability test was conducted in an H-type cell in an alkaline solution.

Table S8. Performance parameters of S_{10}RGO and various reported catalysts.

Catalyst	Electrolyte	j_{total} (mA cm^{-2})	FE (%)	Stability	Production Flow cell (mol g_{cat}^{-1} h^{-1})
S_{10}RGO This work	1 M KOH	50@0.5 V	~90.5	40 h@300 mA cm^{-2}	9.33 ± 0.19
		120@0.27 V	~91	50 h@120 mA cm^{-2}	6.74 ± 0.18
		300@0.15 V	~91.3	50 h@50 mA cm^{-2}	4.28 ± 0.15
		500@ 0.1 V			
N-mFLG-8[22]	1 M KOH	45@0.41 V	~100	50 h@20 mA cm^{-2}	9.66
$OCNS_{900}$[23]	1 M KOH	100@−0.51 V	/	11 h@50 mA cm^{-2}	0.77
CNB-ZIL-8[24]	0.1 M KOH	200@−2.5 V	~80	9 h@40 mA cm^{-2}	1.787
thiophene-S[28]	6 M KOH	/	~92.8	8 h@20 mA cm^{-2}	3.46
B–C[30]	1 M KOH	300@0.28 V	85~90	30 h@200 mA cm^{-2}	/
S-mC-0.375[32]	1 M KOH	220@0.02 V	~95	24 h@185 mA cm^{-2}	25
Co-N-C[33]	0.1 M KOH	/	~70	6 h@50 mA cm^{-2}	4.33
Pb-SA/OSC[37]	1 M KOH	200@0.12 V	~93	100 h@50 mA cm^{-2}	/
Pb-SA/OSC[37]	1 M KOH	200@0.12 V	~92.7	2 h@400 mA cm^{-2}	6.9 mmol cm^{-2} h^{-1}
Co HSACs[45]	1 M KOH	300@0.5 V	~90	25 h@300 mA cm^{-2}	/
NBO-G/CNTs[46]	0.1 M KOH	108@−0.6 V	~80	12 h@50 mA cm^{-2}	0.709
Co-N_5-O-C SACs[37]	1 M KOH	300@0.675 V	~80	24 h@100 mA cm^{-2}	5.92
Co-N_5-O-C SACs[37]	1 M KOH	300@0.675 V	~82.5	24 h@200 mA cm^{-2}	11.3
Sb-NSCF[47]	1 M KOH	120@0.04 V	~80	75 h@50 mA cm^{-2}	7.46

Reference

1 C. C. Su, M. He, R. Amine, Z. Chen and K. Amine, The relationship between the relative aolvating power of electrolytes and shuttling effect of lithium polysulfides in lithium–sulfur batteries, *Angew. Chem. Int. Ed.*, 2018, **57**, 12033-12036.

2 Y. Xu, Y. Wen, Yu. Zhu, K. Gaskell, K. A. Cychosz, B. Eichhorn, K. Xu and C. Wang, C Confined aulfur in microporous carbon eenders superior cycling stability in Li/S batteries, *Adv. Funct. Mater*., 2015, **25**, 4312-4320.

3 H. Sheng, E. D. Hermes, X. Yang, D. Ying, A. N. Janes, W. Li, J. R. Schmidt and S. Jin, Electrocatalytic production of H_2O_2 by selective oxygen reduction using earth-abundant cobalt pyrite (CoS_2), *ACS Catal.*, 2019, **9**, 8433–8442.

4 M. G. Ersozoglu, H. Gursu, M. Gencten, A. S. Sarac and Y. Sahin, A green approach to fabricate binder-free S-doped graphene oxide electrodes for vanadium redox battery, *Int. J. Energy Res*., 2020, **45**, 2126−2137.

5 A. Bagri, C. Mattevi, M. Acik, Y. J. Chabal, M. Chhowalla and V. B. Shenoy, Structural evolution during the reduction of chemically derived graphene oxide, *Nat. Chem*., 2010, **2**, 581–587.

6 Y. Zhang, M. Chu, L. Yang, W. Deng, Y. Tan, M. Ma and Q. Xie, Synthesis and oxygen reduction properties of three-dimensional sulfur-doped graphene networks, *Chem. Commun*., 2014, **50**, 6382–6385.

7 M. Klingele, C. Pham, K. R. Vuyyuru, B. Britton, S. Holdcroft, A. Fischer and S. Thiele, Sulfur doped reduced graphene oxide as metal-free catalyst for the oxygen reduction reaction in anion and proton exchange fuel cells, *Electrochem. Commun*., 2017, **77**, 71–75.

8 H. L. Poh, P. Šimek, Z. Sofer and M. Pumera, Sulfur-doped graphene via thermal exfoliation of graphite oxide in H_2S, SO_2, or CS_2 Gas, *ACS Nano*, 2013, **7**, 5262–5272.

9 R. Sánchez-Salas, E. Muñoz-Sandoval, M. Endo, A. Morelos-Gómez and F. López-Urías, Nitrogen and sulfur incorporation into graphene oxide by mechanical process, *Adv. Eng. Mater.*, 2021, **23**, 2001444.

10 Y. Guo, Z. Zeng, Y. Zhu, Z. Huang, Y. Cui and J. Yang, Catalytic oxidation of aqueous organic contaminants by persulfate activated with sulfur-doped hierarchically porous carbon derived from thiophene, *Appl. Catal. Environ. B*, 2018, **220**,

635−644.

11 Y. Qiu, W. Li, W. Zhao, G. Li, Y. Hou, M. Liu, L. Zhou, F. Ye, H. Li and Z. Wei, High-rate, ultralong cycle-life lithium/dulfur batteries enabled by nitrogen-doped graphene, *Nano Lett.*, 2014, **14**, 4821−4827.

12 Z. Song, M. Wang, Z. Wang, Y. Wang, R. Li, Y. Zhang, C. Liu, Y. Liu, B. Xu and F. Qi, Insights into heteroatom-doped graphene for catalytic ozonation: active centers, reactive oxygen speciesevolution, and catalytic mechanism, *Environ. Sci. Technol.*, 2019, **53**, 5337−5348.

13 J. S. Lim, J. Kim, K.-S. Lee, Y. J. Sa and S. H. Joo, Impact of catalyst loading of atomically dispersed transition metal catalysts on H_2O_2 electrosynthesis selectivity, *Electrochim. Acta*, 2023, **444**, 142031.

14 G. F. Han, F. Li, W. Zou, M. Karamad, J. P. Jeon, S. W. Kim, S. J. Kim, Y. Bu, Z. Fu, Y. Lu, S. Siahrostami and J. B. Baek, Building and identifying highly active oxygenated groups in carbon materials for oxygen reduction to H_2O_2, *Nat. Commun.*, 2020, **11**, 2209.

15 Y. Fang, Y. Fan, K. Xie, W. Ge, Y. Zhu, Z. Qi, Z. Song, H. Jiang and C. Li, Boosting hydrogen peroxide electrosynthesis via modulating the interfacial hydrogen-bond environment, *Angew. Chem. Int. Ed.*, 2023, **62**, e202304413.

16 Y. Fan, Y. Chen, W. Ge, L. Dong, Y. Qi, C. Lian, X. Zhou, H. Liu, Z. Liu, H. Jiang and C. Li, Mechanistic insights into surfactant-modulated electrode–electrolyte interface for steering H_2O_2 electrosynthesis, *J. Am. Chem.Soc.*, 2024, **146**, 7575−7583.

17 W. Sun, L. Tang, W. Ge, Y. Fan, X. Sheng, L. Dong, W. Zhang, H. Jiang and C. Li, Anionic surfactant-modulated electrode-electrolyte interface promotes H_2O_2 electrosynthesis, *Adv. Sci.*, 2024, **11**, 2405474.

18 L. Chen, X. Cui, Y. Wang, M. Wang, R. Qiu, Z. Shu, L. Zhang, Z. Hua, F. Cui and C. Wei, One-step synthesis of sulfur doped graphene foam for oxygen reduction reactions, *Dalton Trans.*, 2014, **43**, 3420−3423.

19 X. Xiao, T. Wang, J. Bai, F. Li, T. Ma and Y. Chen, Enhancing the selectivity of H_2O_2 electrogeneration by steric hindrance effect, *ACS Appl. Mater. Interfaces*, 2018, **10**, 42534−42541.

20 J. S. Lim, J. H. Kim, J. Woo, D. S. Baek, K. Ihm, T. J. Shin, Y. J. Sa and S. H. Joo,

Designing highly active nanoporous carbon H_2O_2 production electrocatalysts through active site identification, *Chem*., 2021, **7**, 3114−3130.

21 L. Han, Y. Sun, S. Li, C. Cheng, C. E. Halbig, P. Feicht, J. L. Hübner, P. Strasser and S. Eigler, In-plane carbon lattice-defect regulating electrochemical oxygen reduction to hydrogen peroxide production over nitrogen-doped graphene, *ACS Catal.*, 2019, **9**, 1283−1288.

22 L. Li, C. Tang, Y. Zheng, B. Xia, X. Zhou, H. Xu and S. Z. Qiao, Tailoring selectivity of electrochemical hydrogen peroxide generation by tunable pyrrolic-nitrogen-carbon, *Adv. Energy Mater.*, 2020, **10**, 2000789.

23 S. Chen, T. Luo, K. Chen, Y. Lin, J. Fu, K. Liu, C. Cai, Q. Wang and H. Li, X. Li, Chemical identification of catalytically active sites on oxygen-doped carbon nanosheet to decipher the high activity for electro-synthesis hydrogen peroxide, *Angew. Chem. Int. Ed.*, 2021, **133**, 16743−16750.

24 Tian, Q. Zhang, L. Thomsen, N. Gao, J. Pan, R. Daiyan, J. Yun, J. Brandt, N. López-Salas, F. Lai, Q. Li, T. Liu, R. Amal, X. Lu and M. Antonietti, Constructing interfacial boron-nitrogen moieties in turbostratic carbon for electrochemical hydrogen peroxide production, *Angew. Chem. Int. Ed.*, 2022, **61**, e202206915.

25 Y. Sun, I. Sinev, W. Ju, A. Bergmann, S. r. Dresp, S. Kühl, C. Spöri, H. Schmies, H. Wang and D. Bernsmeier, Efficient electrochemical hydrogen peroxide production from molecular oxygen on nitrogen-doped mesoporous carbon catalysts, *ACS Catal.*, 2018, **8**, 2844–2856.

26 G. Chen, J. Liu, Q. Li, P. Guan, X. Yu, L. Xing, J. Zhang and R. Che, A direct H_2O_2 production based on hollow porous carbon sphere-sulfur nanocrystal composites by confinement effect as oxygen reduction electrocatalysts, *Nano Res.*, 2019, **12**, 2614–2622.

27 V. Duraisamy, S. Venkateshwaran, R. Thangamuthu and S. M. S. Kumar, Hard template derived N, S dual heteroatom doped ordered mesoporous carbon as an efficient electrocatalyst for oxygen reduction reaction, *Int. J. Hydrogen Energy*, 2022, **47**, 40327–40339.

28 R. R. Xie, C. Cheng, R. Wang, J. Li, E. Zhao, Y. Zhao, Y. Liu, J. Guo, P. Yin and T. Ling, Maximizing thiophene–sulfur functional groups in carbon catalysts for highly

selective H_2O_2 electrosynthesis, *ACS Catal.*, 2024, **14**, 4471–4477.

29 S. Xu, Y. Yu, X. Zhang, D. Xue, Y. Wei, H. Xia, F. Zhang and J. Zhang, Enhanced electron delocalization induced by ferromagnetic sulfur doped C_3N_4 triggers selective H_2O_2 production, *Angew. Chem. Int. Ed.*, 2024, 136, e202407578.

30 Y. Xia, X. Zhao, C. Xia, Z.-Y. Wu, P. Zhu, J. Y. Kim, X. Bai, G. Gao, Y. Hu, J. Zhong, Y. Liu and H. Wang, Highly active and selective oxygen reduction to H_2O_2 on boron-doped carbon for high production rates, *Nat. Commun.*, 2021, **12**, 4225.

31 W. Shen, C. Zhang, X. Wang, Y. Huang, Z. Du, M. Alomar, J. Wang, J. Lv, J. Zhang and X. Lu, Sulfur-doped defective nanocarbons derived from fullerenes as electrocatalysts for efficient and selective H_2O_2 electroproduction, *ACS Mater. Lett.*, 2024, **6**, 17–26.

32 J. Du, Y. Liu, M. Sun, J. Guan, A, Chen and B. Han, Highly selective oxygen electroreduction to hydrogen peroxide on sulfur-doped mesoporous carbon, Angew. Chem. Int. Ed., 2025, e202503385.

33 Y. Sun, L. Silvioli, N. R. Sahraie, W. Ju, J. Li, A. Zitolo, S. Li, A. Bagger, L. Arnarson and X. Wang, Activity–selectivity trends in the electrochemical production of hydrogen peroxide over single-site metal-nitrogen-carbon catalysts, *J. Am. Chem. Soc.*, 2019, **141**, 12372–12381.

34 E. Jung, H. Shin, B. H. Lee, V. Efremov, S. Lee, H. S. Lee, J. Kim, W. Hooch Antink, S. Park, K. S. Lee, S. P. Cho, J. S. Yoo, Y.-E. Sung and T. Hyeon, Atomic-level tuning of Co–N–C catalyst for high-performance electrochemical H_2O_2 production, *Nat. Mater.*, 2020, **19**, 436–442.

35 X. Song, N. Li, H. Zhang, L. Wang, Y. Yan, H. Wang, L. Wang and Z. Bian, Graphene-supported single nickel atom catalyst for highly selective and efficient hydrogen peroxide production, *ACS Appl. Mater. Interfaces,* 2020, **12**, 17519−17527.

36 S. Siahrostami, A. Verdaguer-Casadevall, M. Karamad, D. Deiana, P. Malacrida, B. Wickman, M. Escudero-Escribano, E. A. Paoli, R. Frydendal, T. W. Hansen, I. Chorkendorff, I. E. L. Stephens and J. Rossmeisl, Enabling direct H_2O_2 production through rational electrocatalyst design, *Nat. Mater.*, 2013, **12**, 1137−1143.

37 X. Zhou, Y. Min, C. Zhao, C. Chen, M.-K. Ke, S.-L. Xu, J.-J. Chen, Y. Wu and H.-Q. Yu, Constructing sulfur and oxygen super-coordinated main-group electrocatalysts for

selective and cumulative H_2O_2 production, *Nat. Commun.*, 2024, **15**, 193.

38 W. Zhang, J. W. Choi, S. Kim, T. T. Le, S. Nandy, C.-K. Hwang, S. Y. Paek, A. Byeon, K. H. Chae, S. Y. Lee, S. H. Kim, H. Song, J. Kim, J. Oh, J. W. Lee, S. S. Han and J. M. Kim, Penta nitrogen coordinated cobalt single atom catalysts with oxygenated carbon black for electrochemical H_2O_2 production, *Appl. Catal. B*, 2023, **331**, 122712.

39 H. Gong, Z. Wei, Z. Gong, J. Liu, G. Ye, M. Yan, J. Dong, C. Allen, J. Liu, K. Huang, R. Liu, G. He, S. Zhao and H. Fei, Low-coordinated Co–N–C on oxygenated graphene for efficient electrocatalytic H_2O_2 production, *Adv. Funct. Mater.*, 2022, **32**, 2106886.

40 K. Dong, J. Liang, Y. Wang, Z. Xu, Q. Liu, Y. Luo, T. Li, L. Li, X. Shi, A.M. Asiri, Q. Li, D. Ma and X. Sun, Honeycomb carbon nanofibers: A superhydrophilic O_2-entrapping electrocatalyst enables ultrahigh mass activity for the two-electron oxygen reduction reaction, *Angew. Chem. Int. Ed.*, 2021, **60**, 10583-10587.

41 Y. J. Sa, J. H. Kim and S. H. Joo, Active edge-site-rich carbon nanocatalysts with enhanced electron transfer for efficient electrochemical hydrogen peroxide production, *Angew. Chem. Int. Ed.*, 2019, **58**, 1100–1105.

42 Z. Lu, G. Chen, S. Siahrostami, Z. Chen, K. Liu, J. Xie, L. Liao, T. Wu, D. Lin and Y. Liu, High-efficiency oxygen reduction to hydrogen peroxide catalysed by oxidized carbon materials, *Nat. Catal.*, 2018, **1**, 156−162.

43 Y. Pang, K. Wang, H. Xie, Y. Sun, M.-M. Titirici and G.-L. Chai, Mesoporouscarbon hollow spheres as efficient electrocatalysts for oxygen reduction to hydrogen peroxide in neutral electrolytes, *ACS Catal.*, 2020, **10**, 7434−7442.

44 S. Chen, Z. Chen, S. Siahrostami, D. Higgins, D. Nordlund, D. Sokaras, T. R. Kim, Y. Liu, X. Yan, E. Nilsson, R. Sinclair, J. K. Nørskov, T. F. Jaramillo and Z. Bao, Designing boron nitride islands in carbon materials for efficient electrochemical synthesis of hydrogen peroxide, *J. Am. Chem.Soc.*, 2018, **140**, 7851−7859.

45 W. Fan, Z. Duan, W. Liu, R. Mehmood, J. Qu, Y. Cao, X. Guo, J. Zhong and F. Zhang, Rational design of heterogenized molecular phthalocyanine hybrid single-atom electrocatalyst towards two-electron oxygen reduction, *Nat. Commun.*, 2023, **14**, 1426.

46 M. Fan, Z. Wang, K. Sun, A. Wang, Y. Zhao, Q. Yuan, R. Wang, J. Raj, J. Wu, J. Jiang and L. Wang, N–B–OH site-activated graphene quantum dots for boosting electrochemical hydrogen peroxide production, Adv. Mater., 2023, **35**, 2209086.

47 M. Yan, Z. Wei, Z. Gong, B. Johannessen, G. Ye, G. He, J. Liu, S. Zhao, C. Cui and H. Fei, Sb_2S_3-templated synthesis of sulfur-doped Sb-N-C with hierarchical architecture and high metal loading for H_2O_2 electrosynthesis, *Nat. Commun.*, 2023, **14**, 368.
